# How does insecure attachment lead to paranoia? A systematic critical review of cognitive, affective, and behavioural mechanisms

**DOI:** 10.1111/bjc.12361

**Published:** 2022-02-17

**Authors:** Monica Sood, Katherine B. Carnelley, Katherine Newman‐Taylor

**Affiliations:** ^1^ School of Psychology University of Southampton UK

**Keywords:** attachment, mechanisms, mediators, paranoia, psychosis, review, schizophrenia

## Abstract

**Background:**

The relationship between attachment and paranoia is now well established. There is good theoretical reason and evidence to indicate that attachment style affects cognitive, affective, and behavioural processes which, in turn, contribute to the maintenance of paranoia, but this research has not been integrated. We critically and systematically review research that examines relevant cognitive, affective, and behavioural processes, which may explain how attachment insecurity leads to paranoia and constitute key targets in psychotherapeutic interventions for people with psychosis.

**Method:**

We conducted three systematic searches across six databases (PsycINFO, CINAHL, Medline, Web of Science, Embase, and Google Scholar), from inception to September 2021, to investigate key cognitive, affective, and behavioural processes in the attachment–paranoia association.

**Results:**

We identified a total of 1930 papers and critically reviewed 16. The literature suggests that negative self‐ and other‐beliefs, inability to defuse from unhelpful cognitions, and use of maladaptive emotion regulation strategies mediate the association between attachment insecurity and paranoia in people with psychosis/psychotic experience. Attachment‐secure people with psychosis are more likely to seek help and engage with services than attachment‐insecure people.

**Conclusions:**

Attachment styles impact help‐seeking behaviours in people with psychosis and are likely to influence paranoia via self‐ and other‐beliefs, cognition fusion, and emotion regulation – these candidate mechanisms may be targeted in psychological therapy to improve clinical outcomes for people with psychosis, characterized by paranoia.

**Practitioner points:**

- Insecure attachment is likely to lead to paranoia via negative beliefs about self and others, cognitive fusion, and use of maladaptive emotion regulation strategies. These mechanisms can be targeted in psychotherapeutic interventions for psychosis, such as cognitive behaviour therapy, to improve clinical and recovery outcomes.
- People with psychosis who are attachment‐secure are more likely to seek help and engage with services than those who are attachment‐insecure (particularly avoidant). Attachment style can be assessed to predict service engagement and help‐seeking behaviours in people with psychosis.
- Attachment styles are important predictors of key cognitive, affective, and behavioural processes in people with psychosis. These processes can be assessed and incorporated into individualised formulations, and then targeted in therapy to effect psychotherapeutic change.

## Background

Psychosis refers to clinical diagnoses (e.g., schizophrenia), and psychotic‐type experiences (e.g., paranoia). Cognitive behavior therapy (CBT) is a recommended psychotherapeutic intervention for schizophrenia (National Institute for Health & Care Excellence, 2014), though clinical and recovery outcomes are modest (Jones et al., 2018); for example, meta‐analyses show small effect sizes for CBT in reducing schizophrenia symptoms (Jauhar et al., 2014) and no long‐term benefits in improving quality of life, functioning, and distress (Laws, Darlington, Kondel, McKenna, & Jauhar, 2018). Formulation‐based psychological therapies target key processes hypothesised to contribute to the maintenance of mental distress (Dudley & Kuyken, 2013; Tarrier & Johnson, 2016). Additionally, psychosis research is increasingly focused on developing and testing interventions for specific psychotic experiences, such as paranoia (distressing interpersonal threat beliefs) and auditory hallucinations (e.g., hearing voices) (Berry, Bucci, & Danquah, 2020). It follows that if we are to improve psychological interventions for psychosis, we need to target the processes likely to maintain specific psychotic experiences. This review examines candidate mechanisms that may be targeted in psychological therapy for paranoia – a defining experience of psychosis and common in analogue groups (Freeman et al., 2005).

Attachment theory proposes that the availability and responsiveness of early attachment figures shape *internal working models* (Bowlby, 1969), which underlie *attachment styles* and influence interpersonal cognitions, affect, and behaviour (Ainsworth, Blehar, Waters, & Wall, 1978). When distressed, attachment‐anxious individuals exaggerate distress (*hyperactivate*) to elicit attention from inconsistent caregivers. Attachment‐avoidant individuals suppress distress (*deactivate*) and are compulsively self‐reliant due to rejecting caregivers. Attachment‐secure individuals trust that others will be available and responsive and are confident in their ability to manage distress (Mikulincer & Shaver, 2016).

Attachment insecurity is overrepresented in people with psychosis. In their meta‐analysis, Carr, Hardy, and Fornells‐Ambrojo (2018) found that insecure attachment was twice as prevalent in clinical than non‐clinical psychosis populations. Attachment anxiety and avoidance are associated with severe trauma, poor engagement with services, interpersonal problems, and maladaptive coping in people with psychosis (Gumley, Taylor, Schwannauer, & MacBeth, 2014; Korver‐Nieberg, Berry, Meijer, & de Haan, 2014). Insecure attachment styles have also been associated with paranoia in clinical (Korver‐Nieberg, Berry, Meijer, de Haan, & Ponizovsky, 2015), high‐risk (Russo et al., 2018), and non‐clinical populations (MacBeth, Schwannauer, & Gumley, 2008). An initial review suggested a stronger association between paranoia and attachment avoidance (Berry, Barrowclough, & Wearden, 2007), though recent reviews indicate a stronger association with attachment anxiety (Lavin, Bucci, Varese, & Berry, 2019; Murphy, Goodall, & Woodrow, 2020). There is limited evidence demonstrating associations between paranoia and attachment disorganization, probably due to limited self‐report measures of disorganized attachment until recently (Pollard, Bucci, MacBeth, & Berry, 2020).

Research shows that attachment anxiety and avoidance are more strongly associated with paranoia than hallucinations in non‐clinical (Pickering, Simpson, & Bentall, 2008), clinical psychosis (Wickham, Sitko, & Bentall, 2014), and healthy control groups (Sitko, Varese, Sellwood, Hammond, & Bentall, 2016). Consistent with this, different attachment‐threatening events are associated with particular psychotic‐type experiences. For example, institutional care (Bentall, Wickham, Shevlin, & Varese, 2012) and parental neglect (Sitko, Bentall, Shevlin, O'Sullivan, & Sellwood, 2014) are associated with an increased risk of paranoia specifically. Sitko et al. (2014) found that the association between neglect and paranoia was mediated by attachment anxiety and avoidance, suggesting that interpersonal adversity leads to attachment insecurity which, in turn, predicts paranoia.

Recently, researchers have examined the *causal* impact of attachment using priming (Baldwin, Keelan, Fehr, Enns, & Koh‐Rangarajoo, 1996). Experiments manipulating attachment security show that when individuals with high non‐clinical paranoia are primed to feel safe and secure, their paranoia decreases, whereas when primed to feel suspicious/insecure, paranoia increases (Bullock, Newman‐Taylor, & Stopa, 2016; Newman‐Taylor, Kemp, Potter, & Au‐Yeung, 2017). Correspondingly, preliminary case studies of people with schizophrenia demonstrate that security priming reduces paranoia (Pitfield, Maguire, & Newman‐Taylor, 2020).

**Figure 1:**
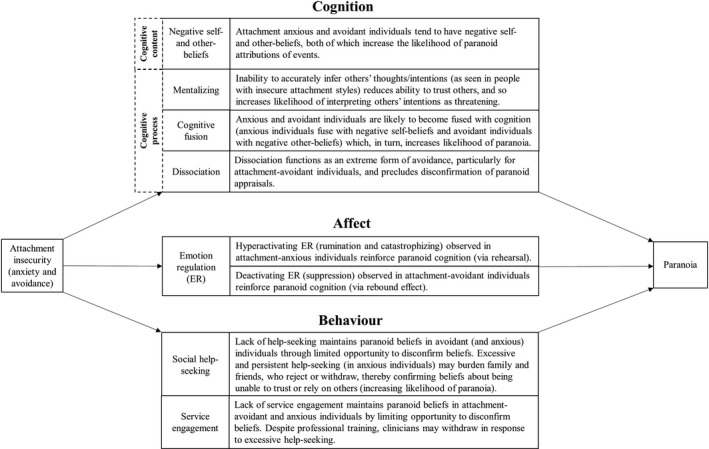
Theoretical model of the mechanisms by which attachment insecurity leads to the development and maintenance of paranoia.

While the association between attachment insecurity and paranoia is now well‐established, the mechanisms in this relationship are poorly understood. There is good theoretical reason and empirical evidence indicating that key cognitive, affective, and interpersonal‐behavioural mechanisms are likely to mediate the attachment–paranoia association. Attachment theory predicts that beliefs about self and others, mentalization, emotion regulation (ER), and help‐seeking are affected by enduring attachment patterns (Ainsworth et al., 1978; Bowlby, 1969). The psychopathology literature indicates that the conceptually linked processes of cognitive fusion and dissociation are also involved in the maintenance of severe mental health problems, such as psychosis and paranoia (Hayes, Strosahl, & Wilson, 2011). We have conceptualized this literature in a theoretical model of how insecure attachment leads to paranoia (Figure 1).

### Beliefs about self and others

Appraisals of ourselves and others – these may be situation‐specific and transient (*automatic thoughts*) or global and stable (*core beliefs*). CBT theorists propose that early experiences lead to the development of beliefs and assumptions about the self, others, and the world, and linked affect, known as *cognitive‐affective schemas*, which guide interpretation of events and subsequent behaviour (Beck, 1967; Young, Klosko, & Weishaar, 2003). Similarly, attachment theorists propose that early attachment experiences lead to the development of internal working models, comprising beliefs about self and others, which guide interpersonal behaviour by operating as templates for future relationships and interactions (Bowlby, 1973). Negative beliefs about self and others are prevalent in people with insecure attachment styles (Bartholomew & Horowitz, 1991) and psychosis (Barrowclough et al., 2003; Krabbendam et al., 2002), and are associated with paranoia in clinical and non‐clinical groups (Bowins & Shugar, 1998; Ellett, Lopes, & Chadwick, 2003; Fowler et al., 2006).

### Mentalizing

Mentalization refers to the implicit and explicit mental processes of inferring one’s own and others’ mental states, such as intentions, beliefs, needs, and feelings (Fonagy & Target, 2002). Attachment theorists propose that our ability to mentalize develops in the context of a secure attachment in which caregivers are consistent, responsive, and frequently mentalizing the child’s internal states. This, in turn, helps the child to understand their own state of mind and infer their caregiver’s intentions (Allen, Fonagy, & Bateman, 2008; Fonagy & Target, 1997, 2002). By contrast, insensitive and inconsistent caregiving interferes with a child’s ability to develop mentalization skills, resulting in impaired mentalization ability among insecurely attached individuals (Fonagy & Target, 1997). Evidence shows that people with psychosis also have mentalization impairments (Harrington, Siegert, & McClure, 2005; O’Driscoll, Laing, & Mason, 2014; Sprong, Schothorst, Vos, Hox, & van Engeland, 2007; Trémeau, 2006).

### Cognitive fusion

Cognitive fusion describes the extent to which we are entangled in our thoughts and beliefs (Hayes et al., 2011), and lies on a continuum from *fused* (thoughts are believed as literally true and dominate behaviour) to *defused* (thoughts are accurately perceived as internal events and do not necessarily impact behaviour) (Gillanders et al., 2014). The literature shows that people with attachment anxiety (Fraley & Shaver, 1997) and paranoia (Stopa, Denton, Wingfield, & Newman‐Taylor, 2013) have easy access to negative cognitions and memories, and once recalled, they become fused with them and find it difficult to stop thinking about them, resulting in increased negative affect.

### Dissociation

Dissociation describes the lack of normal integration of internal experiences, such as thoughts and feelings (Waller & Ross, 1997). Studies show that insecure attachment predicts dissociation (Carlson, 1998; Kong, Kang, Oh, & Kim, 2018; Ogawa, Sroufe, Weinfield, Carlson, & Egeland, 1997) and that dissociation is associated with paranoia (Longden et al., 2020). Attachment avoidance, in particular, is likely to predict dissociation given that dissociation may function as an extreme form of avoidance (e.g., to down‐regulate attachment‐related needs and cognitions) which, in turn, is likely to maintain paranoid beliefs (Figure 1).

### Emotion regulation (ER)

ER describes the ability to manage emotions. Some ER strategies are typically described as ‘adaptive’ because they tend to reduce negative affect and positively impact functioning. Examples include reappraisal (re‐evaluating thoughts and beliefs) and acceptance (having an open attitude towards one’s thoughts and feelings). Others are described as ‘maladaptive’ because they tend to increase negative affect and impair functioning, such as rumination (repetitive rehearsal, e.g., of distressing thoughts), suppression (inhibition of thoughts and emotions), and catastrophization (perceiving a situation as much worse than it is). Securely attached individuals typically use more adaptive ER strategies, whereas insecurely attached individuals typically rely on maladaptive strategies (Mikulincer & Shaver, 2016). Hyperactivating ER strategies used by attachment‐anxious individuals correspond to catastrophization and rumination (Caldwell & Shaver, 2012; Meredith, Strong, & Feeney, 2006), which predict higher levels of paranoia (e.g., Lincoln, Sundag, Schlier, & Karow, 2018). Deactivating ER strategies used by attachment‐avoidant individuals correspond to emotional suppression (Caldwell & Shaver, 2012; Wei, Vogel, Ku, & Zakalik, 2005), which ironically exacerbates distress (known as the rebound effect [Wegner et al., 1987]; Caldwell & Shaver, 2012; Wei et al., 2005), and is therefore likely to reinforce paranoid cognition (cf. Nittel et al., 2018, 2019).

### Help‐seeking

The literature distinguishes *social* help‐seeking (seeking support from one’s social network, e.g., friends and family) and *professional* help‐seeking (seeking help from professionals, e.g., GPs and therapists). Professional help‐seeking is often conceptualized and assessed under the broader umbrella of ‘service engagement’, which refers to a person’s availability for appointments, collaborative responsibility for managing difficulties, help‐seeking (from clinicians), and treatment adherence (Tait, Birchwood, & Trower, 2002). Compared to insecure individuals, secure individuals tend to seek more help and proximity in times of need (Fraley & Shaver, 1998; Simpson, Rholes, & Nelligan, 1992) because they feel confident that attachment figures will be available and responsive. Avoidant individuals, on the other hand, do not typically seek support to maintain a sense of autonomy (Dewitte, Houwer, Buysse, & Koster, 2008; Vogel & Wei, 2005), which may maintain paranoid beliefs by limiting opportunities to disconfirm beliefs. Evidence regarding help‐seeking in attachment‐anxious individuals is inconsistent. Some studies show that they are likely to seek help and do so more than attachment‐avoidant individuals (e.g., Dewitte et al., 2008; Vogel & Wei, 2005), while others suggest that they often do not seek help, perhaps due to fear of rejection and negative perceptions of others’ supportiveness (e.g., Rholes, Simpson, Campbell, & Grich, 2001). Additionally, while attachment‐anxious individuals may seek help, the strategies used may be unhelpful, such that they are insistent and alienating (Adams, Wrath, & Meng, 2018), or indirect (e.g., exaggerating sad facial expressions [Mikulincer & Shaver, 2016]) which others may find burdensome, resulting in withdrawal, and leading to the development/maintenance of paranoid cognition. Poor help‐seeking contributes to the ‘duration of untreated psychosis’ (Birchwood et al., 2013), an international priority of the World Health Organization (WHO, 2018).

Given the robust association between attachment insecurity and paranoia, and the likely role of these cognitive, affective, and interpersonal‐behavioural processes in this association, a review of the literature examining these mechanisms would inform psychological therapies for psychosis that aim to target psychological processes as a means of alleviating distress and facilitating clinical and recovery outcomes.

### Current study

The association between attachment and paranoia is now well‐established (Lavin et al., 2019; Murphy et al., 2020). We sought to extend this literature by critically and systematically synthesizing the research that examines cognitive, affective, and interpersonal‐behavioural mechanisms in this association; this may provide the basis for improving psychotherapies for psychosis by identifying key targets for change. Specifically, we sought to determine whether attachment influences paranoia via cognitive processes (self/other beliefs, mentalization, cognitive fusion, and dissociation), ER strategies, and patterns of help‐seeking/service engagement (Figure 1)^1^, and specify the clinical implications of an integration of this research.

## Method

To obtain a comprehensive account of the literature, we conducted three systematic searches across six databases (PsycINFO, CINAHL, Medline, Web of Science, Embase, and Google Scholar) for published and unpublished literature (Table 1)^2^. All databases were searched from their inception to September 2021 without time or language restrictions.

**Table 1:** Search strategies

	Cognition	Emotion regulation	Help‐seeking
PsycINFO, MEDLINE, CINAHL	attachment AND (psychosis OR psychotic OR schizophreni* OR schizotypy OR paranoi* OR delusion* OR hallucinat*) AND ( (cogniti* OR "core belief*" OR decent* OR mentali* OR schema*) OR ( beliefs N5 (self OR other*) ) )	attachment AND (psychos?s OR psychotic OR schizophreni* OR schizotypy OR paranoi* OR delusion* OR hallucinat*) AND ( ( emotion* N2 (adjust* OR control* OR regulat*) ) OR (affect N2 (adjust* OR control* OR regulat*) ) )	attachment AND (psychos?s OR psychotic OR schizophreni* OR schizotypy OR paranoi* OR delusion* OR hallucinat*) AND ( ( ( behavio* N1 (social OR interpersonal) ) OR ( seek* N1 (help OR support OR proximity) ) OR ("service engagement") ) )
Web of Science	TS = (attachment) AND (psychos?s OR psychotic OR schizophreni* OR schizotypy OR paranoi* OR delusion* OR hallucinat*) AND ((cogniti* OR "core belief*" OR decent* OR mentali* OR schema* OR (beliefs NEAR/5 (self OR other*))))	**TS = **(attachment) AND (psychos?s OR psychotic OR schizophreni* OR schizotypy OR paranoi* OR delusion* OR hallucinat*) AND ((emotion* **NEAR/2** (adjust* OR control* OR regulat*)) OR (affect **NEAR/2** (adjust* OR control* OR regulat*)))	**TS = **(attachment) AND (psychos?s OR psychotic OR schizophreni* OR schizotypy OR paranoi* OR delusion* OR hallucinat*)) AND ((behavio* **NEAR/1** (social OR interpersonal)) OR (seek* **NEAR/1** (help OR support OR proximity) OR “service engagement”))
EMBASE	attachment.**ab,kw,ti**. AND (psychos?s OR psychotic OR schizophreni* OR schizotypy OR paranoi* OR delusion* OR hallucinat*).**ab,kw,ti**. AND (cogniti* OR core belief* OR decent* OR mentali* OR schema* OR belief* about the self OR belief* about other* OR belief* regarding the self OR belief* regarding other* OR self belief*).**ab,kw,ti**.	attachment.**ab,kw,ti**. AND (psychos?s OR psychotic OR schizophreni* OR schizotypy OR paranoi* OR delusion* OR hallucinat*).**ab,kw,ti**. AND (emotion* adjust* OR emotion* control* OR emotion* regulat* OR adjust* emotion* OR regulat* emotion* OR control* emotion* OR adjustment of emotion* OR regulat* of emotion* OR affect* adjust* OR affect control* OR affect regulat* OR adjust* affect OR regulat* affect OR control* affect OR adjustment of affect OR regulat* of affect).**ab,kw,ti**.	attachment.**ab,kw,ti**. AND (psychos?s OR psychotic OR schizophreni* OR schizotypy OR paranoi* OR delusion* OR hallucinat*).**ab,kw,ti**. AND (social behavio* OR interpersonal behavio* OR seek* help OR seek* support OR seek* proximity OR help seek* OR support seek* OR proximity seek* OR service engagement).**ab,kw,ti**.
Google Scholar (first ~200 references)	attachment, psychosis | paranoia | paranoid | hallucination | hallucinate | delusion | schizophrenia | schizophrenic | schizotypy, cognition | mentalization | mentalize | mentalizing | mentalization | belief | ‘core belief	attachment, psychosis | paranoia | paranoid | hallucination | hallucinate | delusion | schizophrenia | schizophrenic | schizotypy, ‘affect regulation’ | ‘emotion regulation’ | affect | emotion	attachment, psychosis | paranoia | paranoid | hallucination | hallucinate | delusion | schizophrenia | schizophrenic | schizotypy, ‘social behaviour’ OR ‘interpersonal behaviour’ OR ‘social behavior’ OR ‘interpersonal behavior’ OR ‘help seeking’ OR ‘support seeking’ OR ‘proximity seeking’ OR ‘help’ OR ‘support’ OR ‘proximity’ OR ‘service engagement’

Studies were included if they reported data from people aged 16 years and above with clinical/non‐clinical psychotic‐type experiences or psychosis diagnoses and examined associations between attachment and: (1) cognition (self/other beliefs [appraisals of self and others], mentalization [ability to infer mental states of self and others], cognitive fusion [extent of entanglement in thoughts and beliefs], and dissociation [disintegration of internal experience]), (2) ER strategies (helpful/unhelpful ways of managing feelings), or (3) help‐seeking/service engagement (social and professional support‐seeking and extent of engagement with clinicians/treatment) (see Table S1 for detailed definitions and descriptions of these mechanisms). Studies were included if they used validated measures of attachment and paranoia. Studies were excluded if: (1) we could not translate them to English and (2) they were reviews, books/chapters, and conference extracts. Studies that did not measure psychotic‐type experience (e.g., paranoia) were only included if participants were people with psychosis or psychotic‐type experience.

Following duplicate removal, two independent reviewers screened titles, abstracts, and full texts to identify eligible studies. Disagreements/uncertainties were resolved through discussion between the two reviewers and, if necessary, with a third reviewer. Eligible full texts and relevant reviews were hand searched to identify further references.

### Quality assessment

Following PRISMA recommendations, we quality assessed studies using the adapted Effective Public Health Practice Project tool (EPHPP; Thomas, 2003). Williams, Bucci, Berry, and Varese (2018) adapted the EPHPP to assess the quality of mediation studies, which are the primary focus of the present review. Using this tool, we rated studies as ‘weak’, ‘moderate’, or ‘strong’ on the following domains: selection bias, confounders, data collection methods (measures), withdrawals/dropouts, and analysis strategy, and also provided a global rating for each study.

## Results

**Figure 2:**
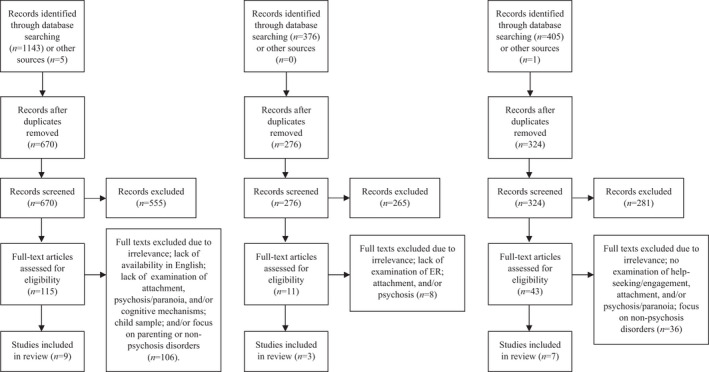
PRISMA diagrams for the cognition (left panel), emotion regulation (middle panel), and help‐seeking (right panel) searches.

Of 1930 records identified (cognition: 1148, ER: 376, help‐seeking: 406) (Figure 2), 16 were reviewed (cognition: nine [Table 2], ER: three [Table 3], help‐seeking: seven [Table 4]; two studies examined multiple mechanisms and were reviewed separately for each). All but one study (Jones, 2015; doctoral thesis) are published in peer‐reviewed journals. Most were conducted in the United Kingdom (*n* = 14) and published between 2011 and 2021 (*n* = 15), indicating the growth of interest in this area over the last decade.

**Table 2:** Data extracted: Cognitive processes

Author(s), date, country	Sample	Design and analysis	Attachment measure(s)	Mechanism assessed (and measure[s] used)	Paranoia/psychosis measure(s)	Main (relevant) findings
Udachina and Bentall (2014), United Kingdom	Students (*N* = 302; 224 females, 71 males, 7 unidentified); Mean age = 22.01	Cross‐sectional; mediation using structural equation modeling (Hu & Bentler, 1998)	Relationship Questionnaire	Negative self‐beliefs (Self‐Esteem Rating Scale‐Short Form)	Persecution and Deservedness Scale	Negative self‐beliefs mediated the association between attachment and paranoia; low attachment security was associated with more negative self‐beliefs (e.g., ‘I am unworthy of love’) and, in turn, more paranoia.
Pickering et al. (2008), United Kingdom	Students (*N* = 503; 350 females, 153 males) aged 18–63 years (*M* = 20.9; *SD* = 5.22)	Cross‐sectional; mediation (Baron & Kenny, 1986)	Relationship Questionnaire	Negative self‐beliefs (Self‐Esteem Rating Scale)	Persecution and Deservedness Scale	Negative self‐beliefs mediated the association between dispositional attachment anxiety and avoidance and non‐clinical paranoia; greater levels of attachment anxiety and avoidance were associated with more negative self‐beliefs and, in turn, more paranoia.
Martinez et al. (2021), United Kingdom	General population (*N* = 1121; 50.7% female) aged 18–86 years (*M* = 47.8; *SD* = 17.2)	Cross‐sectional; mediation using structural equation modeling (Kline, 2015)	Relationship Questionnaire	Negative self‐beliefs (Self‐esteem Rating Scale‐Short Form)	Revised Paranoia and Deservedness Scale	Negative self‐beliefs mediated the association between dispositional attachment anxiety (not avoidance) and trait non‐clinical paranoia; greater levels of attachment anxiety were associated with more negative self‐beliefs and, in turn, more paranoia.
Wickham et al. (2014), United Kingdom	Schizophrenia spectrum disorder diagnosis (inpatient, outpatient, and community) (*N* = 176; 123 males, 53 females); aged 17–77 years	Cross‐sectional; mediation (Preacher & Hayes, 2008)	Relationship Questionnaire	Negative self‐beliefs (Self‐esteem Rating Scale)	Persecution and Deservedness Scale; Positive and Negative Syndrome Scale	Negative self‐beliefs fully mediated the relationship between dispositional attachment anxiety and paranoia, and partially mediated the association between attachment avoidance and paranoia; higher levels of attachment insecurity were associated with more negative self‐beliefs and, in turn, more paranoia.
Sood et al. (2021), United Kingdom^a^	General population sample of adults with high non‐clinical paranoia (*N* = 303; 182 males, 2 non‐binary, 2 unidentified), aged 18–65 years (*M* = 26.24, *SD* = 8.30)	Experimental, two‐part study; mediation (Hayes, 2018)	Manipulated attachment security, anxiety, and avoidance using mental imagery priming Dispositional attachment style measured using the Experiences in Close Relationships Inventory – Short Form	Negative beliefs about self and others (Brief Core Schema Scales) Cognitive fusion (Cognitive Fusion Questionnaire)	Adapted paranoia checklist (state paranoia) Paranoia Scale (trait paranoia)	State cognitive fusion and negative beliefs about self and others mediated the relationship between attachment imagery and state non‐clinical paranoia and anxiety; relative to secure‐primed individuals, attachment‐anxious and ‐avoidant‐primed individuals were more fused with negative cognitions and held more negative self/other beliefs and, therefore, felt more paranoid and anxious.
MacBeth et al. (2011), United Kingdom	People with first‐episode psychosis (*N* = 34; 20 males, 14 females); age at first contact ‐ *M* = 23.32; *SD* = 7.59	Cross‐sectional; Spearman correlations, Mann–Whitney, and Kruskal–Wallis	Adult Attachment Interview	Mentalizing (derived using the Adult Attachment Interview)	Positive and Negative Syndrome Scale	Securely and anxiously attached individuals with psychosis had better mentalizing ability than avoidantly attached people with psychosis; secure and anxious individuals did not differ from each other. Neither attachment nor mentalizing were related to paranoia.
Korver‐Nieberg et al. (2013), United Kingdom^b^	Adolescents who had experienced a psychotic episode (*n* = 32) and controls (*n* = 78) aged 13–18 years	Cross‐sectional; hierarchical multiple regression	Psychosis Attachment Measure	Mentalizing (perspective‐taking task; Dumontheil et al., 2010)	Green Paranoid Thoughts Scale	Perspective‐taking errors were not associated with paranoia or attachment.
Sood and Newman‐Taylor (2020), United Kingdom	Adults with high non‐clinical paranoia (*N* = 117; 84 females, 33 males) aged 18–65 years (*M* = 21.60, *SD* = 6.07)	Experimental, two‐part study; mediation (Hayes, 2018)	Manipulated attachment style: secure vs. insecure/threat Dispositional attachment style measured using the Experiences in Close Relationships Inventory – Short Form	Cognitive fusion (Cognitive Fusion Questionnaire)	Paranoia checklist (state paranoia) Paranoia Scale (trait paranoia)	Cognitive fusion mediated the impact of attachment imagery on paranoia; relative to the insecure/threat imagery group, the secure imagery group was less fused with their negative thoughts and, therefore, less paranoid.
Newman‐Taylor et al. (2021), United Kingdom^a^	Adults with high‐non‐clinical paranoia (*N* = 76; 65 females, 11 males) aged 18–50 years (*M* = 20.53, *SD* = 4.57)	Experimental, longitudinal; mediation (Hayes, 2018)	Manipulated attachment security vs. avoidance Dispositional attachment style measured using the Experiences in Close Relationships Inventory – Short Form	Cognitive fusion (Cognitive Fusion Questionnaire)	Adapted paranoia checklist (state paranoia) Paranoia Scale (trait paranoia)	Cognitive fusion mediated the impact of attachment imagery on paranoia; relative to the avoidant imagery group, the secure imagery group was less fused with their negative thoughts and, therefore, less paranoid.

^a^ These studies examined multiple mechanisms and thus were reviewed separately for each in the narrative synthesis.

^b^ This study was included as the sample comprised people within the target age range specified for this review.

**Table 3:** Data extracted: Emotion regulation

Author(s), date, country	Sample	Design and analysis	Attachment measure(s)	Mechanism assessed (and measure[s] used)	Paranoia/psychosis measure(s)	Main (relevant) findings
Owens et al. (2012), United Kingdom	People with a psychosis diagnosis (*N* = 81; 42 males, 39 females); age – *M* = 38.06, *SD* = 11.55	Cross‐sectional; Pearson’s correlation and hierarchical multiple linear regression	Psychosis Attachment Measure	Emotion Regulation (Difficulties in Emotion Regulation Scale)	Positive and Negative Syndrome Scale	Attachment anxiety and therapeutic alliance (assessed using the Working Alliance Inventory) predicted emotion regulation (while controlling for psychotic experience and negative emotion); anxiously attached individuals with psychosis were likely to react intensely to stress, and those who reported a strong therapeutic alliance had a better understanding of their emotions and were able to use adaptive ER strategies and behave in line with desired goals when upset.
Jones (2015), United Kingdom	Outpatients with first‐episode psychosis and ICD‐10 clinical psychosis diagnosis (*N* = 51; 58.8% male, 41.2% female); age – *M* = 22.45; *SD* = 4.29	Cross‐sectional; mediation (Hayes, 2018)	Relationship Questionnaire	Emotion regulation (Regulation of Emotion Questionnaire)	Positive and Negative Syndrome Scale	Association between attachment security and psychotic‐type experience was mediated by internal‐dysfunctional ER; secure individuals used less internal‐dysfunctional ER (e.g., ‘I keep the feelings locked up inside’) which, in turn, was associated with less psychotic‐type experience. ER did not mediate the association between fearful‐avoidance and psychotic‐type experience broadly and, surprisingly, the relationship between attachment avoidance and psychotic‐type experience was mediated by internal‐functional ER (e.g., ‘I put the situation into perspective’).
Ascone et al. (2020), Germany	People with a psychosis diagnosis (*n* = 60; 63.3% female) and healthy controls (*n* = 40; 67.5% female) Age 18–65 years (psychosis: *M* = 40.2; *SD*=11.7; controls: *M* = 40.1; *SD* = 10.8)	Cross‐sectional; mediation using multiple group structural equation modeling	Relationship Scales Questionnaire	Emotion regulation (Cognitive Emotion Regulation Questionnaire)	Paranoia Checklist	Hyperactivating ER strategies (rumination and catastrophization) mediated the relationship between attachment anxiety (not avoidance) and paranoia in those with psychosis, but not healthy controls; greater use of hyperactivating strategies was associated with higher paranoia in attachment‐anxious individuals with psychosis. Blaming others did not mediate the association between attachment avoidance and paranoia.

**Table 4:** Data extracted: Help‐seeking/service engagement

Author(s), date, country	Sample	Design and analysis	Attachment measure(s)	Mechanism assessed (and measure[s] used)	Paranoia/psychosis measure(s)	Main (relevant) findings
Dozier (1990), United Kingdom	People with schizophrenia and depression diagnoses (*N* = 42) Age 21–60 (*M* = 35) Gender not reported.	Cross‐sectional; correlation	Adult Attachment Interview, classified using the Q‐set/Q‐sort	Clinicians rated participants’ service engagement (i.e., help‐seeking, treatment use, and self‐disclosure). Measure name not provided.	None – examined associations between attachment style and help‐seeking in people with psychosis, but no measure of psychotic‐type experience	Secure individuals relied on attachment figures, in this case, clinicians, and complied with treatment. Avoidant individuals denied needing help, rarely sought help, rejected treatment/support, and tended not to disclose personal problems. Anxious, compared to avoidant, individuals were more expressive about feelings, sought help more often, but were not more compliant with treatment.
Kvrgic et al. (2011), Switzerland	People with a DSM‐IV schizophrenia/ schizoaffective disorder diagnosis (*N* = 127) Aged 18–65 years Gender not reported	Cross‐sectional; correlation	German Psychosis Attachment Measure	Service engagement (Service Engagement Scale)	Positive and Negative Syndrome Scale	Attachment anxiety was positively associated with treatment adherence though there were no associations among other attachment styles and components of service engagement, including help‐seeking.
MacBeth et al. (2011), United Kingdom	People with first‐episode psychosis (*N* = 34; 20 males, 14 females); age at first contact ‐ *M* = 23.32; *SD* = 7.59	Cross‐sectional; Spearman correlations, Mann‐Whitney, and Kruskal–Wallis	Adult Attachment Interview	Service engagement (Service Engagement Scale)	Positive and Negative Syndrome Scale	Secure individuals reported greater treatment adherence than anxious and avoidant individuals, and were better engaged than avoidant individuals, but did not differ from anxious individuals; there were no differences in engagement between attachment‐anxious and ‐avoidant individuals.
Macinnes et al. (2016), United Kingdom	People with schizophrenia (92.2%) or personality disorder diagnoses from medium/high secure psychiatric prisons (*N* = 64; 62 males). Aged 19–67 years (*M* = 42.3; *SD* = 11.9)	Cross‐sectional; multiple regression	Relationship Scales Questionnaire	Service engagement (Service Engagement Scale)	None – examined associations between attachment style and help‐seeking in people with psychosis, but no measure of psychotic‐type experience	Although 73.4% of the sample were insecurely attached, attachment insecurity was not associated with service disengagement.
Newman‐Taylor et al. (2021), United Kingdom	Adults with high‐non‐clinical paranoia (*N* = 76; 65 females, 11 males) aged 18–50 years (*M* = 20.53, *SD* = 4.57)	Experimental, longitudinal; mediation (Hayes, 2018).	Manipulated attachment security vs. avoidance using written primes Dispositional attachment style measured using the Experiences in Close Relationships Inventory – Short Form	Help‐seeking (State Help‐Seeking Measure)	Adapted paranoia checklist (state paranoia) Paranoia Scale (trait paranoia)	Help‐seeking intentions increased in the secure‐primed group across the first three days and from day 3 to 4 in the avoidant‐primed condition. There were no differences in help‐seeking between the secure‐ and avoidant‐primed groups.
Sood et al. (2021), United Kingdom	General population sample of adults with high non‐clinical paranoia (*N* = 303; 182 males, 2 non‐binary, 2 unidentified), aged 18–65 years (*M* = 26.24, *SD* = 8.30)	Experimental, cross‐sectional; mediation (Hayes, 2018)	Manipulated attachment security, anxiety, and avoidance using mental imagery primes Dispositional attachment style measured using the Experiences in Close Relationships Inventory – Short Form	Help‐seeking (State Help‐Seeking Measure)	Adapted paranoia checklist (state paranoia) Paranoia Scale (trait paranoia)	Those primed with secure attachment imagery reported more state help‐seeking intentions than those primed with anxious‐ or avoidant‐imagery; the anxious‐primed group did not differ from the avoidant‐ or secure‐primed groups on help‐seeking.
Tait et al. (2004), United Kingdom	ICD‐10 psychosis diagnosis and history of psychosis episode(s) (*N* = 50) Age and gender not reported	Prospective, longitudinal; one‐sample *t*‐test	Revised Adult Attachment Scale	Service engagement (Service Engagement Scale)	Positive and Negative Syndrome Scale (Structured Clinical Interview)	Insecure attachment styles, compared to secure attachment, were associated with less service engagement.

Most studies were rated as weak in quality (*n* = 10) and only one strong (Table 5). While most studies used validated instruments to assess key variables and appropriate analyses, most were weak due to selection bias (*n* = 14) and not considering and/or covarying potential confounders (*n* = 10).

**Table 5:** Quality assessment ratings

Mechanism Author(s)	Selection bias	Confounders	Data collection ‐ measures			Withdrawals/dropouts	Analysis	Global
			Attachment	Paranoia/Psychosis	Mechanism			
Self/other beliefs								
*Martinez et al. (2021)	W	W	S	M	S	N/A	S	W
*Pickering et al. (2008)	W	W	S	S	S	N/A	M	W
*Sood et al. (2021)	M	S	S^a^	S	S	N/A	S	S
*Udachina and Bentall (2014)	W	W	S	S	S	N/A	S	W
*Wickham et al. (2014)	W	S	S	S	S	N/A	S	M
Mentalizing								
Korver‐Nieberg et al. (2013)	W	S	S	S	S^b^	N/A	S	M
MacBeth et al. (2011)	W	W	S	S	M	N/A	M	W
Cognitive fusion								
*Newman‐Taylor et al. (2021)	W	S	S^a^	S	S	W	S	W
*Sood et al. (2021)	M	S	S^a^	S	S	N/A	S	S
*Sood and Newman‐Taylor (2020)	W	S	S^a^	S	S	N/A	S	M
Emotion regulation								
*Ascone et al. (2020)	W	W	S	S	M	N/A	S	W
*Jones (2015)	M	W	S	S	S	N/A	S	M
Owens et al. (2012)	W	M	S	S	S	N/A	S	M
Help‐seeking/service engagement								
Dozier (1990)	W	S	S	N/A	W	N/A	W	W
Kvrgic et al. (2011)	M	W	S	S	M	N/A	S	M
MacBeth et al. (2011)	W	W	S	S	S	N/A	M	W
Macinnes et al. (2016)	W	W	S	N/A	S	N/A	S	W
*Newman‐Taylor et al. (2021)	W	S	S^a^	S	M	W	S	W
*Sood et al. (2021)	S	S	S^a^	S	M	N/A	S	S
Tait et al. (2004)	M	W	M	N/A	M	M	S	M

Note

W = Weak, M = moderate, S = strong. Studies that examined multiple mechanisms were quality assessed separately, for each mechanism.

Studies marked with an asterisk used mediation analysis.

^a^ Manipulated attachment style using priming and measured trait attachment style using a well‐established, standardized self‐report questionnaire.

^b^ Used a mentalizing task rather than a self‐report questionnaire.

### Synthesis of evidence examining candidate mechanisms in the attachment–paranoia association

#### Beliefs about self and others

Five studies found that negative self‐beliefs mediate the association between insecure attachment and paranoia; higher levels of attachment anxiety and avoidance predicted more negative self‐beliefs which, in turn, predicted higher levels of paranoia (Martinez, Agostini, Al‐Suhibani, & Bentall, 2021; Pickering et al., 2008; Sood, Carnelley, & Newman‐Taylor, 2021; Udachina & Bentall, 2014; Wickham et al., 2014). Most studies found that negative self‐beliefs mediated the association between both attachment anxiety and avoidance and paranoia; just one found that negative self‐beliefs mediated the association between attachment anxiety, but not attachment avoidance, and paranoia (Martinez et al., 2021).

Most studies focused on the mediatory role of negative self‐beliefs. Only one study examined the relative impact of negative beliefs about self and others in the association between primed (not dispositional) attachment and paranoia, and found that primed attachment anxiety and avoidance predicted more negative beliefs about self and others which, in turn, were associated with greater levels of paranoia (Sood et al., 2021).

#### Mentalizing

Only two studies examined associations between mentalization, attachment, and paranoia, and both found no associations between these (Korver‐Nieberg et al., 2013; MacBeth, Gumley, Schwannauer, & Fisher, 2011). The underpowered samples in these studies may have masked the effects; replication with larger clinical samples would determine the reliability of these findings. Korver‐Nieberg et al. (2013) assessed perspective taking, one component of mentalizing, and so other forms of mentalizing (e.g., those based on affective rather than cognitive content) might be associated with paranoia. Interestingly, MacBeth et al. (2011) found that securely and anxiously attached individuals with psychosis had better mentalizing ability than those with an avoidant attachment style – this suggests that there are attachment style differences for mentalizing ability in people with psychosis, but that mentalizing is not related to paranoia specifically.

#### Cognitive fusion

Three recent experimental studies examined the mediatory role of cognitive fusion in the attachment–paranoia association (Newman‐Taylor, Sood, Rowe, & Carnelley, 2021; Sood et al., 2021; Sood & Newman‐Taylor, 2020). Participants completed measures of state cognitive fusion and paranoia before and after random assignment to secure or insecure (anxious/avoidant) attachment priming. In all studies, cognitive fusion mediated the impact of attachment imagery on paranoia; relative to the insecure groups, the secure‐primed groups were less fused with their cognitions and, consequently, less paranoid^3^. The pattern of results was the same in all studies, demonstrating reliability.

#### Dissociation

To our knowledge, no studies have examined dissociation as a mediator in the attachment–paranoia association. The research available suggests that dissociation mediates the association between early adversity and hallucinations (e.g., Berry, Varese, & Bucci, 2017; Varese et al., 2012). There is also evidence that dissociation mediates the *adversity*–paranoia association in non‐clinical and clinical groups (Cole, Newman‐Taylor, & Kennedy, 2016; Mertens, Racioppi, Sheinbaum, Kwapil, & Barrantes‐Vidal, 2021; Pearce et al., 2017), though Perona‐Garcelán et al. (2012) failed to show this.

There is limited evidence of an association between attachment anxiety/avoidance and dissociation (Pearce et al., 2017). Mertens et al. (2021) found that attachment anxiety did not predict dissociation in a non‐clinical student sample, though Kong et al. (2018) found that it did in outpatients with a history of psychological trauma. This suggests that there may be differences in the attachment–dissociation relationship between non‐clinical and clinical groups. However, there is limited evidence showing that attachment *avoidance* predicts dissociation (e.g., Kong et al., 2018; Mertens et al., 2021; Pearce et al. 2017), despite the clear theoretical rationale for this (Figure 1).

#### Emotion regulation (ER)

Only three studies were identified that examined associations between attachment, ER, and psychotic‐type experience (Ascone, Schlier, Sundag, & Lincoln, 2020; Jones, 2015; Owens, Haddock, & Berry, 2012). The results of these studies, overall, suggest that people with secure attachment styles (and strong therapeutic alliances; Owens et al., 2012) are more likely to use adaptive ER strategies which, in turn, are associated with reduced psychotic‐type experience, including paranoia. By contrast, people with insecure attachment styles are more likely to use maladaptive ER strategies (such as rumination), which are likely to reinforce paranoid beliefs (Ascone et al., 2020; Jones, 2015; Owens et al., 2012). Ascone et al. (2020) were the first to demonstrate that hyperactivating ER strategies (rumination and catastrophizing) mediated the association between attachment anxiety (not avoidance) and trait paranoia in people with psychosis, demonstrating that the ER strategies adopted by people with psychosis are congruent with their attachment style.

#### Help‐seeking and service engagement

Seven studies examined associations between attachment and help‐seeking (or service engagement broadly) in people with psychosis (Dozier, 1990; Kvrgic et al., 2011; MacBeth et al., 2011; Macinnes, Macpherson, Austin, & Schwannauer, 2016; Newman‐Taylor et al., 2021; Sood et al., 2021; Tait, Birchwood, & Trower, 2004). Five studies found that people with insecure attachment styles (dispositional or primed) were less likely to engage with services or seek help than those with secure attachment styles (dispositional or primed) (Dozier, 1990; MacBeth et al., 2011; Newman‐Taylor et al., 2021; Sood et al., 2021; Tait et al., 2004); one study found an association between attachment anxiety and treatment adherence, but no other associations (Kvrgic et al., 2011), and one study failed to find any associations between attachment and engagement (Macinnes et al., 2016). Macinnes et al. (2016) propose that this might be because participants were receiving care appropriate to their attachment orientation. However, the study is limited due to its predominantly male sample (only two women) and categorical attachment‐style classification, and the forensic sample prevents generalisation of the findings to other groups (e.g., outpatients).

When distinguishing attachment anxiety and avoidance, two studies found a pattern of results where the engagement/help‐seeking of people with anxious attachment styles fell in between those of secure and avoidant attachment styles (MacBeth et al., 2011; Sood et al., 2021) – attachment‐avoidant individuals were consistently the least likely to seek help and engage. MacBeth et al. (2011) recruited a clinical psychosis sample, and Sood et al. (2021) recruited a non‐clinical, general population sample with high levels of paranoia; the consistent findings across these samples suggest that there are clear attachment‐style differences for help‐seeking and service engagement in people across the non‐clinical–clinical psychosis continuum. However, Newman‐Taylor et al. (2021) failed to find differences in help‐seeking between secure‐ and avoidant‐primed groups (this is likely because the avoidant prime did not have an impact on help‐seeking), though the researchers did find that attachment‐security priming increased help‐seeking intentions, which aligns with the other studies reviewed.

We provide a critical synthesis of this literature in Table 6.

**Table 6:** Critical review and synthesis of study designs, measures, and samples

	Designs	Measures	Samples
Beliefs about self and others	Most studies used cross‐sectional designs and relied on correlational data, precluding causal and temporal inferences (Martinez et al., 2021; Pickering et al., 2008; Udachina & Bentall, 2014; Wickham et al., 2014). Only one study used an experimental design and randomized participants to secure, anxious, or avoidant imagery‐priming conditions (Sood et al., 2021); however, since the mediators (negative beliefs about self and others) were measured (rather than manipulated), we cannot assume that negative beliefs *caused* changes in paranoia. An alternative model in which paranoia predicts attachment insecurity and negative beliefs is not implausible; for example, paranoid beliefs that others are a threat that one cannot manage could elicit negative self/other beliefs which, in turn, may make it difficult to trust others. To infer causation, we need experimental designs that manipulate attachment and beliefs in separate studies (*experimental‐causal‐chain*; Spencer, Zanna, & Fong, 2005).	All studies relied on self‐report assessments and, therefore, would be strengthened through the inclusion of informant, behavioural, or psychophysiological measures. Most studies used the Relationships Questionnaire (Bartholomew & Horowitz, 1991) to measure attachment (Martinez et al., 2001; Picketing et al., 2008; Udachina & Bentall, 2014; Wickham et al., 2014), which comprises only one item to measure each attachment style and therefore may be insufficient to capture the complex working models (i.e., interpersonal cognitions, emotion regulation, and behaviours) that underlie attachment styles. Only one study (Sood et al., 2021) manipulated attachment style and demonstrated that attachment *causally* impacts beliefs and paranoia. Most studies used the self‐esteem rating scale to measure negative self‐beliefs (Martinez et al., 2021; Pickering et al., 2008; Udachina & Bentall, 2014; Wickham et al., 2014), and only one used the Brief Core Schema Scales to measure negative self/other beliefs (Sood et al., 2021). The overall pattern of results across the studies was consistent, demonstrating reliability across measures and methods.	Only one study recruited a clinical sample of people with schizophrenia spectrum diagnoses (Wickham et al., 2014). The remaining study samples were non‐clinical; two recruited student samples (Pickering et al., 2008; Udachina & Bentall, 2014), one recruited a large general population sample (Martinez et al., 2021), and one recruited adults from the general population with high levels of non‐clinical paranoia (Sood et al., 2021). The relatively consistent findings across these samples suggest that the results are reliable across the non‐clinical and clinical psychosis continuum. All but one study (Wickham et al., 2014) had sample sizes of over 300, suggesting that the studies were sufficiently powered for mediation (in line with Kline’s [2005] guidance); the smaller sample in Wickham et al.’s (2014) study is expected given that they recruited a clinical sample.
Mentalizing	Both studies were cross‐sectional, though varied considerably in all other aspects including assessment of attachment, mentalizing, and paranoia. Despite this, both found no associations between these variables.	MacBeth et al. (2011) used the Adult Attachment Interview to capture mentalizing ability, whereas Korver‐Nieberg et al. (2013) used a perspective‐taking task, in which participants were required to actively mentalize. The Adult Attachment Interview minimizes self‐report biases by focusing on participants’ narrative coherence, and mentalizing tasks are useful tools to capture people’s actual (rather than self‐reported) ability to infer mental states of self and others. Both of these methods might therefore be considered more powerful than self‐report measures of mentalizing, which may be problematic given that they require some level of mentalizing (of the self) and metacognition (thinking about thinking) and could be subject to self‐serving biases (e.g., participants may overestimate their ability to understand how they and others feel). Interestingly, no study to date has assessed the association between attachment, paranoia, and self‐reported mentalizing. It would be valuable to see whether the different methods of assessing mentalizing concord with one another. MacBeth’s finding that attachment was not related to paranoia and other psychotic‐type experience is inconsistent with the majority of the literature that shows a strong and consistent association between these variables; therefore, the results might reflect a problem with the study; for example, the researchers categorically classified attachment style rather than viewing these on dimensions. Evidence suggests that categorical measures of attachment lack validity and precision (Fraley & Waller, 1998) because they do not fully capture individual differences in attachment representations; specifically, categorical measures assume that there is no variation among individuals within categories, or that such differences are unimportant (Mikulincer & Shaver, 2016).	Both studies recruited underpowered samples of people with early psychosis and, therefore, it is possible that the underpowered samples masked effects or that mentalizing (or perspective‐taking) impairments develop in the later stages of psychosis.
Cognitive fusion	All studies were experimental. Two used two‐part designs (Part 1 = screening/baseline, Part 2 = experimental manipulation; Sood et al., 2021; Sood & Newman‐Taylor, 2020), and one used a longitudinal design (Newman‐Taylor et al., 2021) and demonstrated a cumulative effect of security priming on state paranoia – this suggests that repeated security priming might not only reduce paranoia, but sustain this effect over time.	All studies used self‐report instruments to assess fusion (Cognitive Fusion Questionnaire) and paranoia (Paranoia Checklist [original or adapted]). The studies manipulated attachment style using priming methods: two used mental imagery primes (Sood et al., 2021; Sood & Newman‐Taylor, 2020), and one used written primes (Newman‐Taylor et al., 2021); the pattern of results was similar across priming methods, demonstrating reliability. Recent systematic reviews show that attachment‐security priming reliably increases several positive outcomes (e.g., positive affect [Rowe, Gold, & Carnelley, 2020]), and suggest that supraliminal priming (e.g., mental imagery) is particularly effective in promoting these benefits (Gillath & Karantzas, 2019).	All studies recruited adults from the general population with high levels of non‐clinical paranoia, and therefore require replication in clinical samples with psychosis. The sample sizes varied considerably across the studies, though were sufficiently powered for the mediation analyses conducted according to Kline’s (2005) recommendations.
Emotion regulation	All studies relied on correlational data and cross‐sectional designs, meaning that we cannot infer causation or temporal precedence.	The studies varied considerably in their assessments of attachment, ER, and paranoia, though all relied largely on self‐report measures; the literature could therefore be extended by incorporating psychophysiological measures, which overcome problems related to self‐report biases and demand characteristics and may help researchers to assess implicit (automatic) ER processes that are unmeasurable using self‐report.	All of the studies recruited people with a psychosis diagnosis; it would be valuable to see whether these effects hold in non‐clinical populations with psychotic‐type experience, to see whether problems with ER are present early and across the psychosis continuum. Given the lack of literature in this area, replications in clinical and non‐clinical samples with paranoia, using longitudinal and experimental designs, are required.
Help‐seeking/service engagement	All but two studies used cross‐sectional designs. One study used a prospective longitudinal design with 3‐ and 6‐month follow‐ups (Tait et al., 2004), and one used a longitudinal design with repeated attachment priming over four days (Newman‐Taylor et al., 2021). Most studies relied on correlations between dispositional attachment style and engagement/help‐seeking; only two studies used experimental designs and manipulated attachment style by priming participants to feel secure or insecure (anxious/avoidant), and measured the impact on help‐seeking (Newman‐Taylor et al., 2021; Sood et al., 2021). Both dispositional and primed secure attachment styles were associated with service engagement and help‐seeking, whereas dispositional/primed attachment anxiety and avoidance were typically associated with service disengagement and lack of help‐seeking (Dozier, 1990; MacBeth et al., 2011; Newman‐Taylor et al., 2021; Sood et al., 2021; Tait et al., 2004), demonstrating consistency across methods and measures.	All studies used self‐report questionnaires of attachment except for Macbeth et al. (2011) who administered the Adult Attachment Interview, which minimizes biases of self‐report techniques by focusing on the coherence of responses. Most studies measured clinician‐rated service engagement/help‐seeking. Little is known about people’s perspectives of their engagement and help‐seeking in other relationships (romantic, peer, family). The Service Engagement Scale assumes that compliance is appropriate; however, people may have good reasons to decline clinical recommendations, which may explain variance in the findings. Future research should examine people’s perceptions of their engagement, and whether these concord with clinician‐rated assessments. Only two studies examined help‐seeking intentions from the person’s perspective (Newman‐Taylor et al., 2021; Sood et al., 2021); however, the help‐seeking measure used did not specify from whom the person would seek help and measured intentions rather than behaviour. Although there is evidence that self‐reported help‐seeking predicts help‐seeking behaviour (Mojtabai, Evans‐Lacko, Schomerus, & Thornicroft, 2016), this literature would benefit from the inclusion of informant/behavioural measures of help‐seeking to determine if self‐reported and clinician‐reported help‐seeking, and help‐seeking intentions and behaviour, are concordant.	One study recruited a first episode psychosis sample (MacBeth et al., 2011); two recruited participants from the general population with high levels of non‐clinical paranoia (Newman‐Taylor et al., 2021; Sood et al., 2021), and the remaining recruited people with psychosis diagnoses (Dozier, 1990; Kvrgic et al., 2011; Macinnes et al., 2016; Tait et al., 2004). The findings suggest that the pattern of results in early psychosis and non‐clinical paranoia samples are similar to those found in established psychosis (Tait et al., 2004) – all are more engaged (or seek more help) if attachment‐secure or ‐anxious, and less if attachment‐avoidant. This suggests that problems related to service engagement (and possibly help‐seeking specifically) are present early on, which strengthens the argument for examining associations between attachment and help‐seeking in people with early/non‐clinical psychosis to target these problems early. Most studies reviewed were conducted in the United Kingdom, a predominantly individualistic culture; more research is needed to examine attachment style differences for service engagement in people with psychosis in collectivistic cultures, where individuals’ help‐seeking attempts may depend upon the needs, beliefs, and desires of the wider group (cf. Lin & Cheung, 1999; Markus & Kitayama, 1991; Mojaverian et al., 2013). In addition, the research should consider potential gender differences in help‐seeking (Seidler et al., 2016; Thompson et al., 2016). Surprisingly, three studies failed to report gender information (Dozier, 1990; Kvrgic et al., 2011; Tait et al., 2004), two had predominantly male samples (MacBeth et al., 2011; Macinnes et al., 2016), one a predominantly female sample (Newman‐Taylor et al., 2021), and only one had roughly equal numbers of males and females (Sood et al., 2021). Future research on help‐seeking in psychosis may recruit even numbers across genders and control for gender in analyses. It would also be valuable to examine help‐seeking attitudes and behaviours of non‐binary/transgender people with psychosis.

## Discussion

We sought to critically and systematically synthesize the literature that examines candidate mechanisms in the attachment–paranoia association. Specifically, we aimed to determine whether this association can be explained by cognitive processes (beliefs about self/others, mentalization, cognitive fusion, and dissociation), ER strategies, and help‐seeking/service engagement, as predicted by attachment theory. The review showed that people with psychosis, and/or paranoia specifically, report heightened distress and psychotic experience due to negative self/other beliefs, an inability to defuse from unhelpful cognitions, and use of maladaptive ER strategies, and that these processes are likely to mediate the attachment–paranoia association. These problems are overrepresented among those with insecure attachment styles, suggesting that attachment insecurity is likely to be a causal factor in the maintenance of paranoia. There are clear attachment style differences for help‐seeking in psychosis, with insecure attachment predicting poor help‐seeking and service disengagement, but there is no evidence that these mediate the attachment–paranoia relationship. There is also no evidence for dissociation and mentalizing as causal mechanisms in the attachment–paranoia association to date.

### Cognitive mechanisms

#### Beliefs

Negative beliefs about self and others mediate the relationship between (dispositional/primed) attachment style and clinical and non‐clinical paranoia. These results align with attachment theory and psychosocial models of psychosis (Garety, Kuipers, Fowler, Freeman, & Bebbington, 2001) which suggest that attachment‐insecure individuals typically develop negative self/other working models, which adversely impact their ability to trust others and may predispose threat‐based interpretations of ambiguous events.

The literature is limited due to a reliance on cross‐sectional designs and correlational data, precluding causal claims. Future research should manipulate beliefs to determine whether these causally impact paranoia. Most studies focused on the mediatory role of negative self‐beliefs. Only one examined the relative impact of negative self/other beliefs in the association between *primed* attachment and paranoia (Sood et al., 2021), though none have examined this, nor the role of positive self/other beliefs, in the association between *dispositional* attachment and paranoia. In the wider attachment literature, attachment anxiety is reliably associated with negative beliefs about the self, attachment avoidance with negative beliefs about others, and attachment security with positive beliefs about self and others (Bartholomew & Horowitz, 1991). Understanding the influence of positive and negative self/other beliefs on paranoia, and whether these differ by attachment style, would be beneficial as interventions could be refined to target specific causal beliefs. For example, if negative self‐beliefs are a stronger predictor of paranoia than negative beliefs about others, targeting negative self‐beliefs and/or enhancing positive self‐beliefs in CBT may be more effective than (the more usual focus on) negative other‐beliefs in reducing paranoia. Similarly, if attachment security decreases negative and increases positive self/other beliefs, enhancing attachment security may be an effective means of facilitating targeted change. Future research should determine the relative impact of positive and negative self/other beliefs in the attachment–paranoia relationship and means of targeting causal beliefs in therapeutic interventions.

#### Mentalizing

While there is good reason for hypothesizing that mentalization plays a role in the attachment–paranoia association, the limited research examining this relationship suggests that mentalizing is not associated with paranoia (Korver‐Nieberg et al., 2013; Macbeth et al., 2011). However, these studies were underpowered and recruited people in the early stages of psychosis and it is possible that mentalizing impairments develop later. Alternatively, it may be that: (1) affective rather than cognitive components of mentalization may be more strongly associated with paranoia (Korver‐Nieberg et al., 2013), (2) cognitive and affective components of mentalization may work together to influence paranoia (Korver‐Nieberg et al., 2013), and/or (3) mentalization is associated with psychosis broadly rather than paranoia specifically. Each of these provides a fruitful area for research to determine the role of mentalization in paranoia/psychosis, which may form the basis of examining mentalization as a causal process in the attachment–paranoia association.

#### Cognitive fusion

Cognitive fusion mediates the primed attachment–paranoia relationship in analogue groups (Newman‐Taylor et al., 2021; Sood et al., 2021; Sood & Newman‐Taylor, 2020), supporting the notion that it is not only the content of our thoughts and beliefs but also our relationship with cognition that predicts distress (Teasdale, 1999). The findings suggest that researchers and clinicians should focus on the role of cognitive *process* (in addition to *content*) in the maintenance of paranoid beliefs. However, all studies were conducted in non‐clinical groups and, thus, the results require replication in clinical groups with psychosis. Additionally, although there is theoretical justification for assuming that cognitive fusion precedes paranoia (Hayes et al., 2011; Stopa et al., 2013), the findings would be strengthened if cognitive fusion was manipulated. Cognitive fusion provides a novel line for future research in attachment and paranoia.

#### Dissociation

No studies examine dissociation in the attachment–paranoia association. Most show that dissociation mediates the association between attachment disorganization^4^ and voice‐hearing (Berry et al., 2017) and some show that dissociation mediates the *adversity*–paranoia association (Pearce et al., 2017). Berry et al. (2017) proposed that voice‐hearing results from dissociative states which develop partly from attachment disorganization. Therefore, it might be that voice‐hearing and paranoia stem from different patterns of attachment insecurity and are associated with distinct cognitive processes (cf. Bentall et al., 2012). For example, attachment disorganization may result in dissociated states which, in turn, lead to hallucinations (Berry et al., 2017), whereas attachment anxiety/avoidance may impact the valence of self/other beliefs and cognitive fusion which predict paranoia (Sood et al., 2021). In line with this, MacBeth et al. (2011) found that ‘organized’ insecure attachment patterns (i.e., attachment anxiety and avoidance) predicted paranoia, whereas a more complex combination of attachment anxiety and avoidance (i.e., attachment disorganization) within the same person predicted hallucinations. Research is needed to examine whether people with organized insecure attachment styles are more susceptible to dissociative states than people with a secure attachment style; this would provide the basis for examining dissociation as a mediator in the attachment–paranoia relationship.

### Emotion regulation (ER)

Maladaptive ER is likely to mediate the association between insecure attachment style and paranoia, and a strong therapeutic alliance promotes the use of adaptive ER in people with psychosis. This suggests that clinicians should target unhelpful ER to attenuate paranoia, and that attachment style should be assessed and targeted in therapy to predict and conceptualize the use of different ER strategies. The evidence reviewed is limited due to a reliance on cross‐sectional and correlational designs; further work is needed to confirm the role of ER in the attachment–paranoia association. In addition, no research has examined the role of suppression in the attachment avoidance–paranoia association, constituting a major gap in the literature. There is strong evidence that attachment avoidance is characterized by emotional suppression (Caldwell & Shaver, 2012), and that suppression is associated with higher levels of paranoia in people with psychosis and healthy populations (Nittel et al., 2018, 2019); a test of the mediatory role of suppression is therefore required in order to permit a comprehensive examination of ER in the attachment–paranoia association.

### Help‐seeking and service engagement

Attachment style influences the likelihood of help‐seeking among those with psychosis; attachment‐secure individuals are more engaged and seek more help than avoidant individuals, but the findings for those who are attachment‐anxious are less consistent and require further investigation. We need to understand the impact of attachment style on help‐seeking so that clinicians can engage people effectively and facilitate access to recommended treatments. This is important because poor help‐seeking contributes to the duration of untreated psychosis (Birchwood et al., 2013), with attendant human, social, and healthcare costs globally (WHO, 2018).

Other determinants of help‐seeking attitudes and behaviour, such as gender and culture, have important implications for research in this area. Collectivistic cultures emphasize social harmony and interdependence, and the needs and goals of the group typically supersede those of the individual (Markus & Kitayama, 1991). By contrast, individualistic cultures emphasize independence and autonomy, and individuals have personal goals that supersede the interests of the group (Markus & Kitayama, 1991). Evidence suggests that people from collectivist cultures exhibit more concerns regarding mental health stigma and, therefore, are less inclined to seek professional help than those in individualistic cultures and, instead, deal with problems related to mental health themselves or using social support (e.g., Lin & Cheung, 1999; Mojaverian, Hashimoto, & Kim, 2013). This suggests that help‐seeking research may not be generalizable cross‐culturally and should be interpreted within its cultural context. Most studies reviewed were conducted in the United Kingdom, a largely individualistic culture, and the results, therefore, require replication in collectivistic cultures.

Studies also show that men are less likely than women to seek professional help (Thompson et al., 2016) which is often related to conventional male gender roles of masculinity (e.g., Seidler, Dawes, Rice, Oliffe, & Dhillon, 2016). Given that a large portion of people diagnosed with schizophrenia are males (Ochoa, Usall, Cobo, Labad, & Kulkarni, 2012), it is important to consider and possibly control for gender when studying help‐seeking in people with psychosis; none of the studies reviewed did this, and some failed to report gender information (see Table 6), indicating an important limitation of existing research.

### Quality implications for future research

Across all mechanisms reviewed, most studies were rated as strong with regard to analysis strategy and instruments used to assess attachment and paranoia, suggesting that we can be confident in the validity of the results. However, most studies received a global weak rating due to selection bias and not considering and/or covarying potential confounders. Greater attention should be allocated to considering potential covariates and selecting representative samples using effective recruitment methods (instead of relying solely on self‐selection and students [for non‐clinical samples] or having clinicians identify participants [for clinical samples]). We recognize that it may be difficult to employ alternative recruitment strategies (for ethical/practical reasons) and control for confounders if the sample is not sufficiently powered (especially for hard‐to‐reach clinical samples); in such cases, researchers should at least consider and report potential covariates and any bias introduced by their sample/recruitment strategy, and interpret the results in light of these limitations.

The studies reviewed relied largely on correlational data and self‐report assessments of attachment, mechanisms, and paranoia. Future research should manipulate the mechanisms identified in this review to ascertain that these are true mediators and appropriate targets in psychotherapeutic interventions for paranoia. Recent experiments have manipulated attachment style using priming, which is an effective means of demonstrating the causal impact of attachment on paranoia and mediating mechanisms. The literature could be extended by incorporating psychophysiological measures, which address the limitations of self‐report assessments (e.g., self‐serving biases and demand characteristics) and enable assessment of implicit processes. Future researchers should also consider self‐reports alongside reports from clinicians and close others (e.g., friends and family), and whether these are concordant.

Although we have described the impact of relevant mechanisms in the attachment–paranoia relationship independently, it is important to acknowledge that these processes are likely to be interrelated and may compound the effects of one another. An analysis of the associations between these processes, and cumulative impacts, would be valuable.

### Conclusions and clinical implications

In conclusion, attachment theory provides a useful framework to conceptualize and predict the role of cognitive, affective, and interpersonal–behavioural processes in the development and maintenance of psychosis, and paranoia specifically (Gumley et al., 2014). This review identifies clear therapeutic targets (see Table 7), derived from attachment theory, which can be incorporated into recommended psychological interventions, such as CBT (for which outcomes remain modest, cf. Jones et al., 2018). Attachment style impacts help‐seeking behaviours in people with psychosis and influences paranoia via self/other beliefs, cognition fusion, and emotion regulation. While many of the recommended interventions may be used in current practice, therapists do not yet consistently consider each of these when planning treatment. The integration of this literature suggests that clinicians should routinely target these cognitive, affective, and behavioural processes to improve clinical outcomes for people with psychosis characterized by paranoia.

**Table 7:** Clinical implications

Mechanisms	Clinical implications
Beliefs about self and others	Assess and target negative self/other beliefs in therapy for paranoia in those with anxious and avoidant attachment styles, given evidence for the mediating role of these beliefs in the relationship between attachment style and paranoia.
Mentalization	No implications from the current review as the limited evidence to date suggests that poor mentalization is not associated with paranoia (despite the clear theoretical argument for this relationship).
Cognitive fusion	Assess and target cognitive fusion (e.g., assess the extent to which individuals believe their paranoid/negative thoughts the and impact of this on their behavior; use defusion exercises to help people ‘step back’ from compelling threat beliefs) because people with paranoia and insecure attachment styles (particularly anxiety) readily access and become fused with negative cognitions and memories, resulting in increased negative affect (and cognitive fusion mediates the relationship between primed insecurity and paranoia).
Dissociation	Assess and target dissociation given the association with paranoia (and disorganized attachment); however, further research is needed given the limited and inconsistent evidence of associations between attachment anxiety/avoidance and dissociation, and lack of evidence examining dissociation as a causal mechanism in the attachment‐paranoia association.
Emotion regulation	Attend to the therapeutic relationship which is likely to act as a secure base from which adaptive ER strategies can be explored. Assess and teach emotion regulation strategies to people who have psychosis and insecure attachment styles. Select particular ER skills for development based on attachment style – for example, practice refocusing as an alternative to rumination for those with anxious attachment, and emotional expression as an alternative to suppression for people with avoidant attachment.
Help‐seeking	Assess and target help‐seeking behaviours for people with psychosis who are insecurely attached; increase help‐seeking and engagement in attachment‐avoidant individuals; assess carefully in those who are attachment‐anxious who may both seek help and struggle to make use of the help they need.
Attachment style	Assess attachment style and use to formulate the development and maintenance of paranoia, including patterns of cognition, emotion regulation, and help‐seeking (i.e., the mechanisms identified in this review, which are likely to vary as a function of attachment style).

Note

Attachment style is not a mechanism but is included in the clinical implications given the results of the review.

## Author contributions

**Monica Sood**: Conceptualization, Methodology, Data curation, Investigation, Formal analysis, Writing – original draft preparation, review, and editing. **Katherine Newman Taylor** and **Katherine Carnelley**: Conceptualization, Methodology, Supervision, Writing – review and editing.

## Conflicts of interest

All authors declare no conflict of interest.

## Funding

This research did not receive any specific grant from funding agencies in the public, commercial, or not‐for‐profit sectors.

## Supporting information


**Table S1**. Glossary of mechanism definitions and associations with attachment and psychosis and/or paranoia.

## Data Availability

Data sharing is not applicable to this article as no new data were created or analysed in this study.

## Footnotes

^1^
A detailed glossary including definitions of these mechanisms and information about how each is theoretically and/or empirically associated with attachment theory and psychosis/paranoia is provided in the Supplementary Material (Table S1).

^2^
The review was not pre‐registered as our aim was to *critically* integrate, for the first time, three bodies of literature directly relevant to psychological therapies for people with psychosis, and examine the evidence for cognitive, affective, and behavioural processes and outcomes through the lens of attachment theory.

^3^
Sood et al. (2021) found that secure‐imagery lead to significantly less paranoia for those relatively moderate and high in attachment anxiety, compared to those with low levels of attachment anxiety. This suggests that secure‐imagery is particularly beneficial for those high in attachment anxiety. By contrast, Hutton et al. (2017) found that secure imagery increased paranoia in attachment‐anxious individuals; however, Hutton et al. (2017) did not use imagery manipulation checks, raising questions about whether the security‐prime induced felt security. Further work is required to examine the impact of security priming for people with insecure attachment styles.

^4^
Some researchers conceptualize disorganized attachment as a mix of attachment anxiety and avoidance, which are ‘organized’ insecure attachment strategies (e.g., Simpson & Rholes, 2002), and others conceptualize it as something that co‐occurs alongside attachment anxiety and avoidance (e.g., Paetzold et al., 2015).
