# Supplementary material for: How does insecure attachment lead to paranoia? A systematic critical review of cognitive, affective, and behavioural mechanisms
Source: Br J Clin Psychol. 2022 Feb 17;61(3):781–815. doi: 10.1111/bjc.12361 (PMC9542899; doi:10.1111/bjc.12361)
Supplement: Supplementary file 1 — Table S1. Glossary of mechanism definitions and associations with attachment and psychosis and/or paranoia. [file BJC-61-781-s001.docx]

Supplementary Material for Manuscript Titled:

*How Does Insecure Attachment lead to Paranoia? A Systematic Critical Review of Cognitive, Affective, and Behavioral Mechanisms*

**Table S1**

*Glossary of Mechanism Definitions and Associations with Attachment and Psychosis and/or Paranoia.*

| **Mechanism** | **Definition** | **Association with Attachment Theory** | **Association with Psychosis/Paranoia** |
| --- | --- | --- | --- |
| Beliefs about self and others | Appraisals of ourselves and others – these may be situation-specific and transient (*automatic thoughts*) or global and stable (*core beliefs*).  Cognitive behavior therapy (CBT) theorists propose that early experiences lead to the development of beliefs and assumptions about the self, others, and the world, and linked affect, known as *cognitive-affective schemas*, which guide behavior (Beck, 1967). In cognitive therapy, schemas refer to organizing principles used to understand or make sense of events and experiences (Beck, 1967; Young et al., 2003). | Attachment theorists propose that early attachment experiences lead to the development of *internal working models*, comprising beliefs about the self, others, and relationships, which guide interpersonal behavior by operating as templates for future relationships and interactions (Bowlby, 1973). For example, when a child is distressed, and their caregiver responds by soothing and comforting them, this leads to a sense that one is worthy of love and others are available and helpfully responsive in times of need. Repeated interactions of this kind result in mental representations of self and others which guide future interpersonal interactions. | Psychosocial models of psychosis hypothesize that early interpersonal experiences and adversities increase vulnerability to negative self- and other-beliefs (Garety et al., 2001).  Negative beliefs about self and others are prevalent in psychosis (Barrowclough et al., 2003; Krabbendam et al., 2002) and paranoia (Bowins & Shugar, 1998; Ellett et al., 2003; Fowler et al., 2006). Barrowclough et al. (2003) found that positive psychotic-type experiences (e.g., delusions) were strongly associated with more negative self-evaluations (even when controlling for depression). Fowler et al. (2006) found that non-clinical paranoia was associated with more negative self- and other-beliefs and fewer positive self- and other-beliefs. |
| Mentalization  (often used interchangeably with ‘theory of mind’) | The implicit and explicit mental processes of inferring one’s own and others’ mental states (e.g., intentions and feelings) (Fonagy & Target, 2002). Three dimensions of mentalization can be identified with this definition (Bateman & Fonagy, 2004):  (a) Implicit (unconscious and automatic) and explicit (conscious and deliberate) operation of mentalization. For example, when therapists try to imagine the mental states of their clients, they do so consciously, whereas turn-taking is typically considered to be unconscious (Allen & Fonagy, 2006).  (b) Cognitive and affective components of mentalization. Mentalizing can be focused on cognitive content (e.g., inferring beliefs and desires) and/or affective content (e.g., empathizing feelings). Cognitive mentalizing tends to be more controlled, whereas affective mentalizing tends to be more automatic.  (c) The object being mentalized: the self and/or other. It is common to mentalize both self and other simultaneously (e.g., couples trying to resolve a conflict may mentalize themselves and their partner to come to a joint understanding).  The concept of mentalization is similar to many others, such as empathy (i.e., implicit and explicit mentalizing of others’ emotions) and mindfulness (i.e., explicit mentalizing of one’s own cognitions and emotions) (Choi-Kain & Gunderson, 2008). | Attachment theorists propose that our ability to mentalize develops in the context of a secure attachment and becomes impaired in the context of insecure attachment (Fonagy & Target, 1997, 2002; Allen et al., 2008). For example, if a parent soothes (contains) their child when distressed, the child can see (a toned-down version of) their experience reflected in their parent’s response, and so learn (a) to calm themselves, and (b) how they feel (because they are observing this in the parent) (Fonagy & Target 1997). If the child is not soothed and sees a highly distressed response, they (a) don’t learn how to calm themselves, and (b) do learn that their feelings are dangerous. If the child’s distress is not recognized or is rejected, they (a) don’t learn how to calm themselves, and (b) do learn to ‘avoid’ or suppress these feelings themselves. Both anxious and avoidant patterns of relating make it difficult for the child to develop an awareness of their feelings as well as others’ perceptions of their feelings and may result in erroneous inferences of the mental states of self and others.  Concerning the development of paranoia, Fonagy and Allison (2014) argue that genuine curiosity and interest of caregivers (and others) in their children as individuals with minds promotes their development of *epistemic trust* (i.e., “openness to the reception of social communication that is personally relevant and of generalizable significance”; Luyten et al., 2020, p. 89) in others, particularly those who treat the child as a meaningful agent. When caregivers accurately represent children as meaningful agents and respond appropriately to their needs, this engenders feelings of security and the child interprets their caregiver’s intentions as caring, enabling them to develop trust in their caregiver (e.g., trust that caregivers will be available in times of need and in the information provided by caregivers). Lack of engagement with the child as a meaningful agent is therefore likely to negatively impact the ability to trust others, increasing the risk of paranoia. That is, when caregivers inconsistently and inaccurately respond to their child’s needs, it becomes difficult for the child to trust that their needs will be met, and they may develop chronic mistrust of others’ messages (Weijers et al., 2020). Furthermore, when caregivers are abusive, trust is likely to be even more detrimentally impacted, and children are more likely to interpret the intentions of others as threatening (paranoia). | People with psychosis experience difficulties in inferring others’ emotions (O’Driscoll et al., 2014) and mental states (Harrington et al., 2005; Sprong et al., 2007), and also experience difficulty verbalizing their own mental states (Trémeau, 2006).  Mentalization-based therapy (MBT) is feasible in psychosis and promotes benefits including increased treatment engagement and social functioning (Brent, 2009, 2015; Lana et al., 2015; Weijers et al., 2016, 2020). MBT assumes that psychosis is partly a problem of poor mentalization and, so, aims to increase people’s capacity to mentalize by helping them to reflect on their current emotional states using questioning and reflection tasks. The MBT therapist aims to prompt the greatest level of mentalization that the person is capable of and takes a therapeutic stance of genuine curiosity, modeled on secure attachment (Pereira & Debanne, 2018; Weijers et al., 2020). This suggests that secure attachments, which promote mentalization ability, may play a protective role in psychosis.  There is clear theoretical reason to assume mentalization deficits in paranoia; inability to understand others’ thoughts and intentions is likely to impact the ability to trust others and increase susceptibility to interpret others’ intentions as threatening. |
| Cognitive fusion | The ability to separate or distance ourselves from our thoughts, and let them come and go, rather than get caught up in them (Gillanders et al., 2014; Hayes et al., 2011). Researchers have proposed a continuum of cognitive fusion from *fused* (thoughts are believed as literally true and dominate behavior), to *defused* (thoughts are accurately viewed as internal events and do not necessarily impact behavior) (Gillanders et al., 2014). | People with attachment anxiety experience difficulty stepping back from negative cognitions and memories (Mikulincer & Orbach, 1995). When anxiously attached individuals are asked to picture their romantic partner leaving them for someone else, they quickly comply and find it difficult to stop thinking about the imagined event, whereas attachment-avoidant individuals are more able to suppress separation-related thoughts (Fraley & Shaver, 1997; Gillath et al., 2005). This suggests that attachment-anxious individuals tend to focus their attention on attachment-related stimuli and often have difficulty disengaging with this information (e.g., Mikulincer et al., 2000; Mikulincer et al., 2002).  People with attachment anxiety (but not avoidance) experience difficulty focusing on the present moment and instead become entangled in their internal experience, which prevents them from engaging in goal-directed behavior (Salande & Hawkins, 2017). | Decreased believability (a proxy for defusion) is associated with reduced distress in people with psychosis (Bach & Hayes, 2002; Gaudiano et al., 2010; Gaudiano & Herbert, 2006).  Cognitive fusion predicts paranoia and paranoia-related distress in a general population and psychosis sample of cannabis users (Newman-Taylor et al., 2020).  Qualitative evidence suggests that people with psychosis (characterized by paranoia) describe being trapped in their thoughts and unable to disengage with their threat beliefs (Stopa et al., 2013). |
| Dissociation | The “lack of normal integration of thoughts, feelings, and experiences into the stream of consciousness and memory*”* (Waller & Ross, 1997, p. 727). Several dissociative processes have been proposed: *absorption* (i.e., becoming absorbed in internal experience [e.g., imagination] or external stimuli [e.g., a book]; Soffer-Dudek, 2018), *depersonalization* (i.e., feeling detached from the self; Holmes et al., 2005), and *derealization* (i.e., feeling that one’s surroundings are not real; Holmes et al., 2005). Some researchers propose that dissociation lies on a continuum of severity ranging from fleeting dissociative states (such as absorption) that are common in the general population (Hunter et al., 2004; Roche & McConkey, 1990), to more distressing experiences (such as dissociative identity disorder) that are seen in clinical populations (Bernstein & Putnam, 1986)*.* Others argue that there are two distinct forms of dissociation: detachment and compartmentalization (see Holmes et al., 2005). | Studies show that disorganized attachment is a developmental predictor of dissociation (Carlson, 1998; Ogawa et al., 1997). Research showing associations between attachment anxiety/avoidance and dissociation is inconsistent (as discussed in the main manuscript). | Meta-analytic evidence shows that dissociation is associated with positive psychotic-type experience, including hallucinations and paranoia (Longden et al., 2020).  There is evidence that dissociation mediates the association between early adversity and psychosis (Anglin et al., 2015; Evans et al., 2015), particularly hallucinations (Berry et al., 2017, 2018; Varese et al., 2012). |
| Emotion  Regulation (ER) | The ability to manage emotions by redirecting them in a desired direction (Gross, 2007; Koole, 2009).  Some ER strategies are identified as ‘adaptive’ because they tend to positively impact functioning, reduce negative affect, and increase positive affect. Examples include cognitive reappraisal (i.e., re-evaluating thoughts and beliefs) and acceptance (i.e., having an open attitude toward one’s thoughts and feelings). Others are identified as ‘maladaptive’ because they tend to impair functioning and increase negative affect, such as rumination (i.e., repetitive rehearsal, e.g., of distressing thoughts), suppression (i.e., inhibition of thoughts and emotions), catastrophization (i.e., perceiving a situation as significantly worse than it actually is), and self- or other-blaming. | Relative to insecurely attached individuals, secure individuals tend to be more confident in their ability to manage distress, show more control over negative emotions, tend not to catastrophize in negative situations (Meredith et al., 2006), and use more adaptive ER strategies such as reappraisal, self-soothing, and help-seeking (Mikulincer & Shaver, 2019).  By contrast, people with insecure attachment patterns use more maladaptive and less adaptive ER strategies and find it difficult to identify and describe their emotions. Anxiously attached individuals tend to *hyperactivate* when distressed. In adults, hyperactivation corresponds to emotional reactivity (i.e., feeling overwhelmed by negative emotions and not being able to control them; Wei et al., 2005), catastrophization (Meredith et al., 2006), and rumination (Caldwell & Shaver, 2012; Garrison et al., 2014; Reynolds et al., 2014).  Those with avoidant attachment styles tend to *deactivate* when distressed. In adults, deactivation corresponds to emotional suppression; Caldwell and Shaver (2012) and Wei et al. (2005) found that suppression mediates the association between attachment avoidance and negative mood, meaning that emotional suppression among avoidant individuals ironically exacerbates distress (known as the rebound effect; Wegner et al., 1987). Garrison et al. (2014) found that suppression mediates the association between attachment avoidance and disclosure of distress, suggesting that avoidant individuals who suppress their emotions are less likely to share their distress with others. | Whereas adaptive ER is crucial to mental wellbeing, maladaptive ER is associated with psychopathology cross-diagnostically (Aldao et al., 2012; Kring & Werner, 2004).  There is evidence that hyperactivating and deactivating ER strategies are used by people with psychosis, who typically struggle to regulate their emotion and use fewer adaptive, and more maladaptive ER strategies (e.g., Kimhy et al., 2012; Lincoln et al., 2015a; 2015b; Livingstone et al., 2009; O’Driscoll et al., 2014; Perry et al., 2011).  Hyperactivating and deactivating ER strategies have also been observed in people with paranoia (or are associated with paranoia) in clinical, high-risk, and non-clinical groups. Lincoln et al. (2018) found that following social exclusion, maladaptive ER strategies (i.e., catastrophizing, rumination, self- and other- blaming) predicted increased paranoia, whereas adaptive ER strategies (i.e., reappraisal, acceptance, and refocusing) predicted attenuated paranoia, in clinically high-risk of psychosis participants. Nittel et al. (2018, 2019) found that expressive suppression predicted increased subclinical paranoia in those with psychosis and non-clinical controls. |
| Help-seeking / service engagement | The literature distinguishes two forms of help-seeking:   - Social help-seeking: seeking help from one’s social network, such as friends and family. - Professional help-seeking: seeking support from professionals, such as GPs or therapists.   Help-seeking has been measured using different methods. Self-reported help-seeking measures a person’s help-seeking attitudes and intentions or typical patterns of help-seeking behavior, whereas observable help-seeking involves the examination of overt behavior. Researchers have also examined informant estimations of help-seeking (e.g., a clinician rates a client’s help-seeking behavior).  Service engagement is conceptualized as a person’s availability for appointments, collaborative responsibility for managing difficulties, help-seeking (from clinicians), and treatment adherence (Tait et al., 2002). | Compared to insecure individuals, secure individuals tend to seek more help and proximity in times of need (behavioral observations: Fraley & Shaver, 1998; Simpson et al., 1992; self-report: Larose et al., 1999; Ognibene & Collins, 1998), because they feel confident that attachment figures will be available and responsive to their needs.  Avoidant individuals, on the other hand, do not typically seek support in order to maintain a sense of autonomy (Dewitte et al., 2008; Pascuzzo et al., 2013; Vogel & Wei, 2005).  Evidence regarding help-seeking in anxiously attached individuals is inconsistent. Some evidence shows that attachment-anxious individuals are quite likely to seek help and do so more than attachment-avoidant individuals (Dewitte et al., 2008; Karantzas & Cole, 2011; Kruger & Djerf, 2015; Vogel & Wei, 2005). Other studies suggest that attachment-anxious individuals often do not seek help (e.g., Rholes et al., 2001). One explanation for these inconsistent findings is that while anxiously attached individuals crave intimacy and protection from others, they also fear rejection. Anxiously attached individuals often have negative perceptions of others’ supportiveness (e.g., Rholes et al., 2001), which may lead to less help-seeking (e.g., Karantzas & Cole, 2011). Additionally, while attachment-anxious individuals may seek help, their help-seeking strategies may be unhelpful, such that they are insistent and alienating (Adams et al., 2018), or indirect (e.g., anxious individuals often exaggerate sad facial expressions) which others may find irritating (Mikulincer & Shaver, 2016). | There is often a treatment delay following first-episode psychosis (termed duration of untreated psychosis; DUP), indicating a lack of help-seeking (Birchwood et al., 2013; World Health Organisation, 2018). Research shows a long DUP is partly accounted for by the delay within mental health services and poor help-seeking (Birchwood et al., 2013). The ‘access and waiting times standard’ to implement early intervention in psychosis addresses mental health service delays (NICE, 2016). However, poor help-seeking has not been addressed. Research also shows that people with paranoia have poor help-seeking behaviors (Melo & Bentall, 2010).  A common method of examining help-seeking in psychosis is by measuring service engagement, conceptualized as availability for appointments, collaborative responsibility for managing difficulties, help-seeking, and treatment adherence (Tait et al., 2002). A systematic review found that approximately 30% of people with first-episode psychosis disengage from services despite ongoing clinical needs (Doyle et al., 2014). Service disengagement hinders social, interpersonal, and vocational recovery (Addington & Gleeson, 2005). Among the reasons for poor help-seeking and service disengagement, significant others and beliefs about service providers seem to play a particularly important role. That is, people with psychosis typically seek professional help with support from significant others (Anderson et al., 2013; Cadario et al., 2012; Doyle et al., 2014; Boydell et al., 2006; Judge et al., 2005), and the maintenance of help-seeking is predicted, at least partly, by the level of trust in services (Smith et al., 2013). Since support from significant others and trust are key factors determining attachment style, it follows that the quality of attachments (outside and within services) is likely to be an important determinant of help-seeking in psychosis. |

**References**

Adams, G. C., Wrath, A. J., & Meng, X. (2018). The relationship between adult attachment and mental health care utilization: A systematic review. *Canadian Journal of Psychiatry*, *63*(10), 651–660. https://doi.org/10.1177/0706743718779933

Addington, J., & Gleeson, J. (2005). Implementing cognitive-behavioural therapy for first-episode psychosis. *British Journal of Psychiatry, 187*(s48), s72–s76. https://doi.org/10.1192/bjp.187.48.s72

Aldao, A. (2012). Emotion regulation strategies as transdiagnostic processes: A closer look at the invariance of their form and function. *Revista de Psicopatología y Psicología Clínica, 17*(3), 261–277. https://doi.org/10.5944/rppc.vol.17.num.3.2012.11843

Allen, J. G., Fonagy, P., & Bateman, A. W. (2008). *Mentalizing in clinical practice*. Washington, DC: American Psychiatric Publishing.

Allen, J. G., & Fonagy, P. (Eds.). (2006). The handbook of mentalization-based treatment. John Wiley & Sons Inc. [https://doi.org/10.1002/9780470712986](https://psycnet.apa.org/doi/10.1002/9780470712986)

Anderson, K. K., Fuhrer, R., & Malla, A. K. (2013). “There are too many steps before you get to where you need to be”: Help-seeking by patients with first-episode psychosis. *Journal of Mental Health, 22*(4), 384–395. https://doi.org/10.3109/09638237.2012.705922

Anglin, D. M., Polanco-Roman, L., & Lui, F. (2015). Ethnic variation in whether dissociation mediates the relation between traumatic life events and attenuated positive psychotic symptoms. *Journal of Trauma and Dissociation, 16*(1), 68–85. https://doi.org/10.1080/15299732.2014.953283

Bach, P., & Hayes, S. C. (2002). The use of acceptance and commitment therapy to prevent rehospitalization of psychotic patients: a randomized controlled trial. *Journal of Consulting and Clinical Psychology, 70*(5), 1129–1139. https://doi.org/10.1037//0022-006x.70.5.1129

Barrowclough, C., Tarrier, N., Humphreys, L., Ward, J., Gregg, L., Andrews, B. (2003). Self-esteem in schizophrenia: relationships between self-evaluation, family attitudes, and symptomatology. *Journal of Abnormal Psychology, 112*(1), 92–99. https://doi.org/[10.1037/0021-843X.112.1.92](https://www.researchgate.net/deref/http%3A%2F%2Fdx.doi.org%2F10.1037%2F0021-843X.112.1.92?_sg%5B0%5D=ceChcbHensbKcI88zqx-PqG6JJW5LxhcjpL2wBIgH3GRrj25LpjmZHuB488q6aqq00Ht0jqmYKLb9y2sA8lePTqbdQ.vPO-slbwTzy3-yPM2eYcVg_hXDqwHUM2BksOkDQamfsg_PXav5m5z-89k22dAATQclXld_7ELSCtzIj9VYDbjA)

Bateman, A. W. and Fonagy, P. (2004). *Psychotherapy for Borderline Personality Disorder: mentalization based treatment.* Oxford: Oxford University Press.

Beck, A.T. (1967). *Depression.* Harper and Row: New York.

Bernstein, E., & Putnam, F. W. (1986). Development, reliability and validity of a dissociation scale. *Journal of Nervous and Mental Disease, 174*(12), 727–735. https://doi.org/10.1097/00005053-198612000-00004

Berry, K., Fleming, P., Wong, S., & Bucci, S. (2018). Associations between trauma, dissociation, adult attachment and proneness to hallucinations. *Behavioural and Cognitive Psychotherapy, 46*(3), 292–301. https://doi.org/10.1017/S1352465817000716

Berry, K., Varese, F., & Bucci, S. (2017). Cognitive attachment model of voices: evidence base and future implications. *Frontiers in Psychiatry*, *8*. https://doi.org/10.3389/fpsyt.2017.00111

Birchwood, M., Connor, C., Lester, H., Patterson, P., Freemantle, N., Marshall, M., Fowler, D., Shôn, L., Jones, P., Amos, T., Everard, L., & Singh, S. P. (2013). Reducing duration of untreated psychosis: care pathways to early intervention in psychosis services. *British Journal of Psychiatry, 203*(1), 58–64. https://doi.org/10.1192/bjp.bp.112.125500

Bowins, B., & Shugar, G. (1998). Delusions and self-esteem. *Canadian Journal of Psychiatry, 43*(2), 154–158. https://doi.org/10.1177/070674379804300204

Bowlby, J. (1973). *Attachment and loss: Separation* (Vol. 2). Basic Books.

Boydell, K. M., Gladstone, B. M., & Volpe, T. (2006). Understanding help seeking delay in the prodrome to first episode psychosis: A secondary analysis of the perspectives of young people. *Psychiatric Rehabilitation Journal, 30*(1), 54–60. https://doi.org/10.2975/30.2006.54.60

Brent, B. (2009). Mentalization-based psychodynamic psychotherapy for psychosis. *Journal of Clinical Psychology, 65*(8), 803–814. https://doi.org/10.1002/jclp.20615

Brent, B. K., & Fonagy, P. (2015). A mentalization-based treatment approach to disturbances of social understanding in schizophrenia. In P. Lysaker, G. Dimaggio, & M. Brune (Eds.), *Social Cognition and Metacognition in Schizophrenia: Psychopathology and Treatment Approaches* (pp. 245-259). USA: Elsevier

Cadario, E., Stanton, J., Nicholls, P., Crengle, S., Wouldes, T., Gillard, M., & Merry, S. M. (2012). A qualitative investigation of first-episode psychosis in adolescents. *Clinical Child Psychology and Psychiatry, 17*(1), 81–102. https://doi.org/10.1177/1359104510391860

Caldwell, J. G., & Shaver, P. R. (2012). Exploring the cognitive-emotional pathways between adult attachment and ego- resiliency. *Individual Differences Research, 10*(3), 141–152.

Carlson, E. A. (1998). A prospective longitudinal study of attachment disorganization/disorientation. *Child Development*, *69*(4), 1107–1128. https://doi.org/10.1111/j.1467-8624.1998.tb06163.x

Choi-Kain, L. W., & Gunderson, J. G. (2008). Mentalization: ontogeny, assessment, and application in the treatment of borderline personality disorder. *American Journal of Psychiatry, 165*(9), 1127–1135. https://doi.org/10.1176/appi.ajp.2008.07081360

Dewitte, M., Houwer, J., Buysse, A., & Koster, E. H. W. (2008). Proximity seeking in adult attachment: Examining the role of automatic approach-avoidance tendencies. *British Journal of Social Psychology, 47*(4), 557–573. https://doi.org/10.1348/014466607x265148

Doyle, R., Turner, N., Fanning, F., Brennan, D., Renwick, L., Lawlor, E., & Clarke, M. (2014). First-episode psychosis and disengagement from treatment: A systematic review. *Psychiatric Services, 65*(5), 603–611. https://doi.org/10.1176/appi.ps.201200570

Ellett, L., Lopes, B., & Chadwick, P. (2003). Paranoia in a nonclinical population of college students. *The Journal of Nervous and Mental disease*, *191*(7), 425–430. https://doi.org/10.1097/01.NMD.0000081646.33030.EF

[Evans, G. J](https://www.ncbi.nlm.nih.gov/pubmed/?term=Evans%20GJ%5BAuthor%5D&cauthor=true&cauthor_uid=26099655)., [Reid, G](https://www.ncbi.nlm.nih.gov/pubmed/?term=Reid%20G%5BAuthor%5D&cauthor=true&cauthor_uid=26099655)., [Preston, P](https://www.ncbi.nlm.nih.gov/pubmed/?term=Preston%20P%5BAuthor%5D&cauthor=true&cauthor_uid=26099655)., [Palmier-Claus, J](https://www.ncbi.nlm.nih.gov/pubmed/?term=Palmier-Claus%20J%5BAuthor%5D&cauthor=true&cauthor_uid=26099655)., & [Sellwood, W](https://www.ncbi.nlm.nih.gov/pubmed/?term=Sellwood%20W%5BAuthor%5D&cauthor=true&cauthor_uid=26099655). (2015). Trauma and psychosis: The mediating role of self-concept clarity and dissociation. *Psychiatry Research, 228*(3), 626–632. https://doi.org/10.1016/j.psychres.2015.04.053

Fonagy, P., & Allison, E. (2014). The role of mentalizing and epistemic trust in the therapeutic relationship. Psychotherapy, 51(3), 372–380.  https://doi.org/10.1037/a0036505

Fonagy, P., & Target, M. (1997). Attachment and reflective function: Their role in self-organization. *Development and Psychopathology, 9*(4), 679–700. https://doi.org/10.1017/S0954579497001399

Fonagy, P., & Target, M. (2002). Early intervention and the development of self-regulation. *Psychoanalytic Inquiry, 22*(3), 307–335. https://doi.org/10.1080/07351692209348990

Fowler, D., Freeman, D., Smith, B., Kuipers, E., Bebbington, P., Bashforth, H., Coker, S., Hodgekins, J., Gracie, A., Dunn, G., & Garety, P. (2006). The Brief Core Schema Scales (BCSS): psychometric properties and associations with paranoia and grandiosity in non-clinical and psychosis samples. *Psychological Medicine*, *36*(6), 749–759. https://doi.org/10.1017/S0033291706007355

Fraley, R. C., & Shaver, P. R. (1997). Adult attachment and the suppression of unwanted thoughts. *Journal of Personality and Social Psychology, 73*(5), 1080–1091. https://doi.org/10.1037/0022-3514.73.5.1080

Fraley, R. C., & Shaver, P. R. (1998). Airport separations: A naturalistic study of adult attachment dynamics in separating couples. *Journal of Personality and Social Psychology, 75*(5), 1198–1212. https://doi.org/10.1037/0022-3514.75.5.1198

Garety, P., Kuipers, E., Fowler, D., Freeman, D., & Bebbington, P. (2001). A cognitive model of the positive symptoms of psychosis. *Psychological Medicine*, *31*(02), 189–195. https://doi.org/10.1017/s0033291701003312

Garrison, A. M., Kahn, J. H., Miller, S. A., & Sauer, E. M. (2014). Emotional avoidance and rumination as mediators of the relation between adult attachment and emotional disclosure. *Personality and Individual Differences, 70*, 239–245. https://doi.org/10.1016/j.paid.2014.07.006 0191-8869

Gaudiano, B. A., Herbert, J. D. (2006). Believability of hallucinations as a potential mediator of their frequency and associated distress in psychotic inpatients. *Behavioural and Cognitive Psychotherapy, 34*(4), 497–502. https://doi.org/10.1017/S1352465806003080

Gaudiano, B. A., Herbert, J. D., & Hayes, S. C. (2010). Is it the symptom of the relation to it? Investigating potential mediators of change in acceptance and commitment therapy for psychosis. *Behavior Therapy*, *41*(4), 543–554. https://doi.org/10.1016/j.beth.2010.03.001

Gillanders, D. T., Bolderston, H., Bond, F. W., Dempster, M., Flaxman, P. E., Campbell, L., Kerr, S., Tansey, L., Noel, P., Ferenbach, C., Masley, S., Roach, L., Lloyd, J., May, L., Clarke, S., & Remington, B. (2014). The development and initial validation of the cognitive fusion questionnaire. *Behavior Therapy, 45*(1), 83–101. https://doi.org/10.1016/j.beth.2013.09.001

[Gillath, O](https://www.ncbi.nlm.nih.gov/pubmed/?term=Gillath%20O%5BAuthor%5D&cauthor=true&cauthor_uid=16087352)., [Bunge, S. A](https://www.ncbi.nlm.nih.gov/pubmed/?term=Bunge%20SA%5BAuthor%5D&cauthor=true&cauthor_uid=16087352)., [Shaver, P. R](https://www.ncbi.nlm.nih.gov/pubmed/?term=Shaver%20PR%5BAuthor%5D&cauthor=true&cauthor_uid=16087352)., [Wendelken, C](https://www.ncbi.nlm.nih.gov/pubmed/?term=Wendelken%20C%5BAuthor%5D&cauthor=true&cauthor_uid=16087352)., & [Mikulincer, M](https://www.ncbi.nlm.nih.gov/pubmed/?term=Mikulincer%20M%5BAuthor%5D&cauthor=true&cauthor_uid=16087352). (2005). Attachment-style differences in the ability to suppress negative thoughts: exploring the neural correlates. *NeuroImage, 28*(4), 835–847. https://doi.org/10.1016/j.neuroimage.2005.06.048

Gross, J. J. (Ed.). (2007). Handbook of emotion regulation. Guilford Press.

Harrington, L., Siegert, R., & McClure, J. (2005). Theory of mind in schizophrenia: A critical review. *Cognitive Neuropsychiatry, 10*(4), 24. 249–286. https://doi.org /10.1080/13546800444000056

Hayes, S. C., Strosahl, K. D., & Wilson, K. G. (2011). *Acceptance and Commitment Therapy: The process and practice of mindful change* (2nd ed.). New York: Guilford.

Holmes, E. A., Brown, R. J., Mansell, W., Fearon, R. P., Hunter, E. C., Frasquilho, F., & Oakley, D. A. (2005). Are there two qualitatively distinct forms of dissociation? A review and some clinical implications. *Clinical Psychology Review, 25*(1), 1–23. https://doi.org/10.1016/j.cpr.2004.08.006

Hunter, E. C. M., Sierra, M., & David, A. S. (2004). The epidemiology of depersonalisation and derealisation: A systematic review. *Social Psychiatry and Psychiatric Epidemiology, 39*(1), 9–18. https://doi.org/10.1007/s00127-004-0701-4

Judge, A. M., Perkins, D. O., Nieri, J., & Penn, D. L. (2005). Pathways to care in first episode psychosis: A pilot study on help-seeking precipitants and barriers to care. *Journal of Mental Health, 14*(5), 465–469. https://doi.org/10.1080/09638230500271089

Karantzas, G. C., & Cole, S. F. (2011). Arthritis and support seeking tendencies: The role of attachment. *Journal of Social and Clinical Psychology, 30*(4), 404–440. https://doi.org/10.1521/jscp.2011.30.4.404

Kimhy, D., Vakhrusheva, J., Jobson-Ahmed, L., Tarrier, N., Malaspina, D., &, Gross, J. J. (2012). Emotion awareness and regulation in individuals with schizophrenia: Implications for social functioning. *Psychiatry Research, 200*(2-3), 193–201. https://doi.org/10.1016/j.psychres.2012.05.029

Koole, S. (2009). The psychology of emotion regulation: an integrative review. *Cognition and Emotion, 23*(1), 4–41. https://doi.org/10.1080/02699930802619031

Krabbendam, L., Janssen, I., Bak, M., Bijl, R. V., de Graaf, R., van Os, J. (2002). Neuroticism and low self-esteem as risk factors for psychosis. *Social Psychiatry and Psychiatric Epidemiology, 37*(1), 1–6. https://doi.org/10.1007/s127-002-8207-y

Kruger, D. J., & Djerf, J. M. (2016). High Ringxiety: Attachment Anxiety Predicts Experiences of Phantom Cell Phone Ringing. *Cyberpsychology, behavior and social networking*, *19*(1), 56–59. https://doi.org/10.1089/cyber.2015.0406

Lana, F., Marcos, S., Cusi, L.M., Vilar, A., Victor, P. S., Romero, M., & Marti-Bonany, J. (2015). Mentalization based group psychotherapy for psychosis: A pilot study to assess safety, acceptance and subjective efficacy. *International Journal of Psychology and Psychoanalysis, 1*, 1–6. https://doi.org/10.23937/2572-4037.1510007

Larose, S., Bernier, A., Soucy, N., & Duchesne, S. (1999). Attachment style dimensions, network orientation and the process of seeking help from college teachers. *Journal of Social and Personal Relationships, 16*(2), 225–247. https://doi.org/10.1177/0265407599162006

Lincoln, T. M., Hartmann, M., Köther, U., & Moritz, S. (2015a). Dealing with feeling: Specific emotion regulation skills predict responses to stress in psychosis. *Psychiatry Research, 228*(2), 216–222. https://doi.org/10.1016/j.psychres.2015.04.003

Lincoln, T. M., Hartmann, M., Köther, U., & Moritz, S. (2015b). Do people with psychosis have specific difficulties regulating emotions? *Clinical Psychology and Psychotherapy, 22*(6), 637–646. https://doi.org/10.1002/cpp.1923

Lincoln, T. M., Sundag, J., Schlier, B., & Karow, A. (2018). The relevance of emotion regulation in explaining why social exclusion triggers paranoia in individuals at clinical high risk of psychosis. *Schizophrenia Bulletin, 44*(4), 757–767. https://doi.org/10.1093/schbul/sbx135

Livingstone, K., Harper, S., & Gillanders, D. (2009). An exploration of emotion regulation in psychosis. *Clinical Psychology and Psychotherapy, 16*(5), 418–430. https://doi.org/10.1002/cpp.635

Longden, E., Branitsky, A., Moskowitz, A., Berry, K., Bucci, S., & Varese, F. (2020). The relationship between dissociation and symptoms of psychosis: A metaanalysis. *Schizophrenia Bulletin*. Advance online publication. https://doi.org/10.1093/schbul/sbaa037

Luyten, P., Campbell, C., & Fonagy, P. (2020). Borderline personality disorder, complex trauma, and problems with self and identity: A social-communicative approach. *Journal of personality*, *88*(1), 88–105. https://doi.org/10.1111/jopy.12483

Melo, S. S., & Bentall, R. P. (2010). Coping in subclinical paranoia: A two nations study. *Psychology and Psychotherapy: Theory, Research and Practice, 83*(4), 407–420. https://doi.org/10.1348/147608310X487542

Meredith, P. J., Strong, J., Feeney, J. A. (2006). The relationship of adult attachment to emotion, catastrophizing, control, threshold and tolerance, in experimentally-induced pain. *Pain, 120*(1-2), 44–52. https://doi.org/10.1016/j.pain.2005.10.008

Mikulincer, M., Birnbaum, G., Woddis, D., & Nachmias, O. (2000). Stress and accessibility of proximity-related thoughts: exploring the normative and intraindividual components of attachment theory. *Journal of Personality and Social Psychology, 78*(3), 509–523. https://doi.org/10.1037//0022-3514.78.3.509

Mikulincer, M., Gillath, O., & Shaver, P. R. (2002). Activation of the attachment system in adulthood: Threat-related primes increase the accessibility of mental representations of attachment figures. Journal of Personality and Social Psychology, 83(4), 881–895. https://doi.org/10.1037/0022-3514.83.4.881#

Mikulincer, M., & Orbach, I. (1995). Attachment styles and repressive defensiveness: The accessibility and architecture of affective memories. Journal of Personality and Social Psychology, 68(5), 917–925.  https://doi.org/10.1037/0022-3514.68.5.917

Mikulincer, M., & Shaver, P. R. (2016). *Attachment in adulthood: Structure, dynamics, and change, 2^nd^ Edition*. Guilford Press.

Mikulincer, M., & Shaver, P. R. (2019). Attachment orientations and emotion regulation. *Current Opinion in Psychology*, *25*, 6–10. https://doi.org/10.1016/j.copsyc.2018.02.006

National Institute for Health and Care Excellence (NICE, 2016). Implementing the early intervention in psychosis access and waiting time standard: Guidance. Retrieved from https://www.nice.org.uk/guidance/cg178/resources/implementing-the-early-intervention-in-psychosis-access-and-waiting-time-standard-guidance-pdf-2487749725

Newman-Taylor, K., Richardson, T., Sood, M., Sopp, M., Perry, E., & Bolderston, H. (2020). Cognitive mechanisms in cannabis-related paranoia. Part 1: Initial testing in a general population sample. *Psychosis, 12*(4) 314–327. https://doi.org/10.1080/17522439.2020.1757742.

Nittel, C. M., Lamster, F., Rief, W., Kircher, T., Soll, D., & Mehl, S. (2019). Emotional instability and expressive suppression are related to paranoia in daily life: An electronic mobile assessment study in nonclinical individual. *Journal of Experimental Psychopathology, 10*(3), 1–16. https://doi.org/10.1177/2043808719868119

Nittel, C. M., Lincoln, T. M., Lamster, F., Leube, D., Rief, W., Kircher, T., & Mehl, S. (2018). Expressive suppression is associated with state paranoia in psychosis: An experience sampling study on the association between adaptive and maladaptive emotion regulation strategies and paranoia. *British Journal of Clinical Psychology, 57*, 291–312. https://doi.org/10.1111/bjc.12174

O’Driscoll, C., Laing, J., & Mason, O. (2014). Cognitive emotion regulation strategies, alexithymia and dissociation in schizophrenia, a review and meta-analysis. *Clinical Psychology Review, 34*(6), 482–495. https://doi.org/10.1016/j.cpr.2014.07.002

Ogawa, J. R., Sroufe, L. A., Weinfield, N. S., Carlson, E. A., & Egeland, B. (1997). Development and the fragmented self: longitudinal study of dissociative symptomatology in a nonclinical sample. *Development and Psychopathology*, *9*(4), 855–879. https://doi.org/10.1017/s0954579497001478

Ognibene, T. C., & Collins, N. L. (1998). Adult attachment styles, perceived social support and coping strategies. *Journal of Social and Personal Relationships, 15*(3), 323–345. https://doi.org/10.1177/0265407598153002

Pascuzzo, K., Cyr, C., & Moss, E. (2013). Longitudinal association between adolescent attachment, adult romantic attachment, and emotion regulation strategies. *Attachment & Human Development*, *15*(1), 83–103. https://doi.org/10.1080/14616734.2013.745713

Pereira, J. G., & Debbane, M. (2018). *An Integrative-Relational Approach in Schizophrenia: From Philosophical Principles to Mentalization-Based Practice*. In: Hipólito, I., Gonçalves, J., and Pereira, J. G., (Eds.), **Schizophrenia and Common Sense. Explaining the Relation Between Madness and Social Values.** (pp. 193-207). Switzerland: Springer, Cham.

Perry, Y., Henry, J. D., & Grisham, J. R. (2011). The habitual use of emotion regulation strategies in schizophrenia. *British Journal of Clinical Psychology, 50*(2), 217–222. https://doi.org/10.1111/j.2044-8260.2010.02001.x

Reynolds, S., Searight, H. R., & Ratwik., S. (2014). Adult attachment style and rumination in the context of intimate relationships. *North American Journal of Psychology*, 16(3), 485–496.

Rholes, W. S., Simpson, J. A., Campbell, L., & Grich, J. (2001). Adult attachment and the transition to parenthood. *Journal of Personality and Social Psychology, 81*(3), 421–435. https://doi.org/10.1037/0022-3514.81.3.421

Roche, S. M., & McConkey, K. (1990). Absorption: Nature, assessment, and correlates. *Journal of Personality and Social Psychology, 59*(1), 91–101. https://doi.org/10.1037/0022-3514.59.1.91

Salande, J. D., & Hawkins, R. C. II. (2017). Psychological flexibility, attachment style, and personality organization: Correlations between constructs of differing approaches. *Journal of Psychotherapy Integration, 27*(3), 365–380. https://doi.org/10.1037/int0000037

Simpson, J. A., Rholes, W. S., & Nelligan, J. S. (1992). Support seeking and support giving within couples in an anxiety-provoking situation: The role of attachment styles. *Journal of Personality and Social Psychology, 62*(3), 434–446. https://doi.org/10.1037/0022-3514.62.3.434

Smith, T. M., Easter, A., Pollock, M., Pope, L. G., & Wisdom, J. P. (2013). Disengagement from care: Perspectives of individuals with serious mental illness and of service providers. *Psychiatric Services, 64*(8), 770–775. https://doi.org/10.1176/appi.ps.201200394

Soffer-Dudek, N. (2018). Dissociative absorption, mind‐wandering, and attention‐deficit symptoms: Associations with obsessive‐compulsive symptoms. *British Journal of Clinical Psychology, 58*(1), 51–69. https://doi.org/10.1111/bjc.12186

Sprong, M., Schothorst, P., Vos, E., Hox, J., & van Engeland, H. (2007). Theory of mind in schizophrenia: meta-analysis. *British Journal of Psychiatry, 191*, 5–13. https://doi.org/10.1192/bjp.bp.107.035899

Stopa, L., Denton, R., Wingfield, M., & Newman-Taylor, K. (2013). The fear of others: a qualitative analysis of interpersonal threat in social phobia and paranoia. *Behavioural and Cognitive Psychotherapy, 41*(1), 188–209. https://doi.org/10.1017/S1352465812000422

Tait, L., Birchwood, M., & Trower, P. (2002). A new scale (SES) to measure engagement with community mental health services. *Journal of Mental Health, 11*(2), 191–198. https://doi.org/10.1080/09638230020023570-2

Trémeau, F. (2006). A review of emotion deficits in schizophrenia. *Dialogues in Clinical Neuroscience, 8*(1), 59–70.

Varese, F., Smeets, F., Drukker, M., Lierverse,, R., Lataster, T., Viechtbauer, W., … Bentall, R. P. (2012). Childhood adversities increase the risk of psychosis: a meta-analysis of patient-control, prospective- and cross-sectional cohort studies. *Schizophrenia Bulletin, 38*, 661-671. https://doi.org/10.1093/schbul/sbs050

Vogel, D. L., & Wei, M. (2005). Adult attachment and help-seeking intent: The mediating roles of psychological distress and perceived social support. *Journal of Counseling Psychology, 52*(3), 347–357. https://doi.org/10.1037/0022-0167.52.3.347

Waller, N. G., & Ross, C. A. (1997). The prevalence and biometric structure of pathological dissociation in the general population: taxometric and behavior genetic findings. *Journal of abnormal psychology*, *106*(4), 499–510. https://doi.org/10.1037//0021-843x.106.4.499

Wegner, D. M., Schneider, D. J., Carter, S. R., & White, T. L. (1987). Paradoxical effects of thought suppression. *Journal of Personality and Social Psychology, 53*(1), 5–13. https://doi.org/10.1037/0022-3514.53.1.5

Wei, M. F., Vogel, D. L., Ku, T. Y., & Zakalik, R. A. (2005). Adult attachment, affect regulation, negative mood, and interpersonal problems: The mediating roles of emotional reactivity and emotional cutoff. *Journal of Counseling Psychology, 52*(1), 14–24.  https://doi.org/10.1037/0022-0167.52.1.14

Weijers, J., ten Kate, C., Eurelings-Bontekoe, E. H. M., Viechtbauer, W., Rampaart, R., Bateman, A., & Selten, J. (2016). Mentalization-based treatment for psychotic disorder: protocol of a randomized controlled trial. *BMC Psychiatry, 16*(191). https://doi.org/10.1186/s12888-016-0902-x

Weijers, J., ten Kate, C., Debbané, M., Bateman, A. W., de Jong, S., Selten, J., -P., C., J., & Eurelings-Bontekoe, E. H. M. (2020). Mentalization and psychosis: A rationale for the use of mentalization theory to understand and treat non‑affective psychotic disorder. *Journal of Contemporary Psychotherapy*. https://doi.org/10.1007/s10879-019-09449-0

World Health Organization. (2018). The ICD-11 classification of mental and behavioural disorders: Clinical descriptions and diagnostic guidelines. World Health Organization.

Kring, A. M., & Werner, K. H. (2004). Emotion Regulation and Psychopathology. In P. Philippot & R. S. Feldman (Eds.), The regulation of emotion (pp. 359–385). Lawrence Erlbaum.

Young, J. E., Klosko, J. S., Weishaar, M. (2003). *Schema therapy: A practitioner’s guide*. Guilford Publications.
